# An fMRI examination of the role of the Locus Coeruleus in state regulation in ADHD

**DOI:** 10.1162/IMAG.a.1200

**Published:** 2026-04-13

**Authors:** Leonhard H. Drescher, Julie M. Hall, Joshua O. Eayrs, Ruth M. Krebs, C. Nico Boehler, Jan R. Wiersema

**Affiliations:** Department of Experimental Clinical and Health Psychology, Ghent University, Ghent, Belgium; University Psychiatric Center KU Leuven, Kortenberg, Belgium; Department of Health, Medical, and Neuropsychology, Leiden University, Leiden, The Netherlands; Liverpool John Moores University, Liverpool, United Kingdom; Department of Experimental Psychology, Ghent University, Ghent, Belgium

**Keywords:** ADHD, fMRI, state regulation, Locus Coeruleus, event rate

## Abstract

The state regulation deficit account of attention-deficit hyperactivity disorder (ADHD) posits that symptoms and performance deficits associated with ADHD are context-dependent and explained by a deficit in arousal regulation. Research into this topic has often used event rate manipulations to induce different arousal states, and has demonstrated deficits at both overstimulating and understimulating event rate levels. Although existing research has provided strong support for the state regulation deficit account, little is known about the neurobiological substrate of state regulation deficits. An important candidate brain network is the Locus Coeruleus-noradrenergic (LC-NE) system, which has been hypothesized by several researchers to play a key role in state regulation deficits in ADHD. In the current study, we examined, for the first time, the role of the LC in state regulation deficits in ADHD using high-resolution fMRI scans. We presented a target detection task at three event rate levels (fast, moderate, slow) to adults with (*n* = 27) and without ADHD (*n* = 28), with 20-s resting intervals at the start and the middle of each event rate condition. No group difference was found for performance, whereas results indicated significantly higher self-reports of state regulation deficits in daily life in the ADHD group. Anatomically guided region-of-interest analyses based on a high-resolution turbo-spin-echo anatomical scan of the pons region indicated an overall lower LC activity during resting intervals in the ADHD group, irrespective of event rate. Event-related LC activity was not impacted by event rate or by group. Our results, therefore, support the notion of a general “underarousal” in ADHD, but do not confirm a relationship between LC activity and behavior, raising doubts on a direct implication of the LC-NE system in state regulation deficits in ADHD.

## 1 Introduction

Attention-deficit hyperactivity disorder (ADHD) is a neurodevelopmental disorder with symptoms of inattention, impulsiveness, and hyperactivity that cause impairment in social, academic, and professional contexts (American Psychiatric Association, 2022; Sonuga-Barke et al., 2022). The worldwide prevalence of ADHD is estimated at 5–7%, and at about 2.6% for adults specifically (Polanczyk et al., 2014; Song et al., 2021; Willcutt, 2012).

ADHD is associated with performance deficits on a wide range of experimental tasks (Alderson et al., 2013; Barkley, 1997; Kofler et al., 2013). It should, however, be stressed that these deficits have been found to be highly context-dependent (Sonuga-Barke et al., 2010). The state regulation deficit account, an influential theoretical account of ADHD, proposes that symptoms and performance deficits in ADHD arise from a difficulty in the regulation of internal energetic states (Johnson et al., 2009; Sergeant, 2000; van der Meere, 2005). This account is based on a long line of research applying the Cognitive Energetic Model by Sanders (1983) in ADHD research. The Cognitive-Energetic Model postulates that (1) stimulation from the context influences the internal arousal level and that (2) performance is best during moderate arousal levels. In other words, according to the Cognitive-Energetic Model, the relationship between performance and arousal follows an inverted U-shaped curve, where poor performance is associated with hypoarousal and hyperarousal, and with arousal, in turn, being modulated by the level of external stimulation. The Cognitive-Energetic Model further postulates that effortful regulation of the internal arousal state is required to optimize performance when the internal arousal state is suboptimal. In addition, this model makes a distinction between phasic and tonic components of arousal: the phasic “arousal” component is defined as a momentary physiological response to sensory input, and the tonic “activation” component fluctuates slowly and represents readiness for (motor) action (Pribram & McGuinness, 1975). According to the state regulation deficit account, ADHD is associated with difficulties in regulating non-optimal levels of the tonic arousal component (i.e., activation).

Research into this topic started in the 1980s with experiments by Sergeant and van der Meere (Sergeant, 2000; Sergeant & van der Meere, 1990), who investigated the potential relationships between different energetic pools and ADHD by systematically manipulating various parameters. They found that performance in ADHD was especially susceptible to manipulations of the pace of the task (i.e., the event rate), which has been argued to affect the activation state. Follow-up research consistently confirmed that event rate had a more pronounced impact on the performance of individuals with ADHD, which corresponds to the interpretation of a deficient regulation of the activation state (Benikos & Johnstone, 2009; Börger & van der Meere, 2000; Metin et al., 2014; Scheres et al., 2001; Wiersema, van der Meere, Antrop, et al., 2006; Wiersema, van der Meere, Roeyers, et al., 2006). The findings indicate that performance deficits are specifically apparent, or more pronounced, during fast and slow event rates, and absent or less pronounced during a moderate event rate (for a meta-analysis, see Metin et al., 2012). It is of note that similar findings have been observed for adults with elevated self-reported ADHD symptomatology (Drescher et al., 2021, 2023; Mohamed et al., 2016a, 2016b), in accord with a dimensional view on ADHD (McLennan, 2016). Some psychophysiological studies have likewise manipulated event rate to investigate arousal regulation difficulties in ADHD. While more such research is needed and despite the low specificity of single physiological measures, the combined findings, indeed, support the notion of arousal regulation difficulties in ADHD, linked to insufficient compensatory effort allocation. In a study by Börger & van der Meere (2000), children with ADHD showed less heart rate deceleration before the onset of Go-signals, particularly in the slow event rate condition, indicating lower readiness to respond (i.e., lower activation). In addition, they exhibited a higher heart rate variability (of the 0.10 Hz frequency component), argued to reflect reduced effort allocation (Börger et al., 1999; Jorna, 1992; Mulder, 1986; but see also Nickel & Nachreiner, 2003). The amplitude of the event-related potential component P3b, also argued to be an index of effort allocation, has been found to be smaller in children as well as adults with ADHD during slow event rates (Wiersema, van der Meere, Antrop, et al., 2006; Wiersema, van der Meere, Roeyers, et al., 2006). Phasic pupil size, another proposed index of effort (Eckstein et al., 2017; Hoeks & Levelt, 1993; Mulder, 1986; Polt, 1970; van der Wel & van Steenbergen, 2018), was found to be smaller in children with ADHD during a fast event rate condition, suggestive of less effort allocation during conditions that elicit suboptimal activation levels (Metin et al., 2017). Note that here, we specifically focused on studies that manipulated event rate to test the state regulation deficit account of ADHD. While a comprehensive review of all relevant research is beyond the scope of this paper, it is worth mentioning that evidence from other research lines investigating peripheral indices of functioning of the autonomic nervous system, such as cortisol, salivary alpha-amylase, and electrodermal activity, have also indicated altered autonomic functioning and arousal in ADHD, which has increasingly supported the view that arousal dysregulation is a core characteristic of ADHD (for reviews, see Bellato et al., 2020, 2023).

Despite the ample amount of behavioral support and the growing literature on psychophysiological correlates of state regulation deficits in ADHD, their neurobiological basis remains largely unknown. Several researchers have pointed to the Locus Coeruleus (LC) as one potential candidate (Bellato et al., 2020; Howells et al., 2012; Imeraj et al., 2012; Konrad et al., 2006; Rowe et al., 2005; Sonuga-Barke et al., 2010; van der Meere, 2005). This may not be surprising, as the LC has been tightly linked to arousal regulation (Aston-Jones & Cohen, 2005; Berridge & Waterhouse, 2003; Poe et al., 2020; Sara & Bouret, 2012). The LC is a small nucleus in the brainstem and the main source of norepinephrine (NE) in the brain, and it is linked to arousal-related functions and wakefulness. While a large body of previous research has focused on dopaminergic activity in the brain in relationship to ADHD, both the dopamine and the NE system have been found to be strongly implicated in the pathophysiology of ADHD (Cortese et al., 2012; del Campo et al., 2011; Konrad et al., 2006). This is unsurprising, as the dopamine and NE systems are intrinsically linked, including the fact that dopamine is the chemical precursor of NE in the brain. In addition, the high effectiveness of atomoxetine, a selective NE reuptake inhibitor, in the treatment of ADHD, points to the key role of NE pathways in the pathophysiology of ADHD. While classically associated with arousal, changes in LC activity are also associated with other functions including motivation, emotion, attention, decision-making, working memory, response inhibition, learning, and motor processes, which implies that it plays a crucial role in the optimization of behavioral performance (Aston-Jones & Cohen, 2005; Aston-Jones & Waterhouse, 2016; Poe et al., 2020; Sara & Bouret, 2012; Unsworth & Robison, 2017).

The LC has two distinct modes of firing: *tonic activity* is the sustained, state-related level of firing that fluctuates slowly over time, and *phasic activity* that consists of momentary, transient spikes with a duration of hundreds of milliseconds (Aston-Jones & Cohen, 2005). Especially stimuli that are salient (i.e., behaviorally relevant—such as target trials in a detection task) are known to elicit a phasic LC response (Krebs et al., 2013; Vazey et al., 2018). Interestingly, the adaptive gain theory of LC function posits that tonic and phasic LC activity are related to each other following an inverted U-shaped function: phasic bursts of firing are more pronounced during moderate levels of tonic activity, and less pronounced during relatively low or high tonic activity (Aston-Jones & Cohen, 2005). According to the adaptive-gain theory, this inverted U-curve relationship relates to behavioral performance in a similar fashion as the classic Yerkes-Dodson principle, that states that performance and arousal follow an inverted U-curve with optimal performance occurring during moderate arousal levels (Winton, 1987; Yerkes & Dodson, 1908). This inverted U-shaped effect of the LC corresponds to the relationship between performance and tonic arousal in the state regulation deficit account. Interestingly, early literature on the Cognitive-Energetic Model already hypothesized an association between the LC and effort allocation in the context of arousal regulation (Mulder, 1986; Sanders, 1983). It should be noted here, however, that although the theoretical link is intriguing, the evidence supporting the assumption of a U-shaped relationship between tonic and phasic LC activity is scarce, with evidence to the contrary existing as well (Hayat et al., 2020).

Dysfunction of the LC may thus be a plausible explanation for state regulation deficits in ADHD, given its key role in attention in general, and specifically in the regulation of arousal. Interestingly, (tonic) LC activity also fluctuates with the circadian rhythm and plays a role in the regulation of the sleep/wake cycle, which, in turn, is disturbed in a large proportion of the ADHD population (Imeraj et al., 2012). Finally, the LC is argued to be involved in the switching between distinct attentional networks (i.e., the Fronto-Parietal Network and the Default Mode Network; Mather et al., 2016; Unsworth & Robison, 2017; Zerbi et al., 2019), which, in turn, has been found to be disrupted in ADHD (Sidlauskaite et al., 2014, 2016b). Indeed, the two existing fMRI studies in ADHD with an event rate manipulation found ADHD-related deviant activity in the thalamus, the anterior cingulate cortex, and the Default Mode Network, which all share strong links with the LC (Kooistra et al., 2010; Metin et al., 2015).

Given the strong plausibility of the role of the LC in state regulation deficits in ADHD, it may seem surprising that as of yet, no fMRI study has attempted to test this. However, it should be noted that assessing LC activity is generally a challenging undertaking due to its small size and location in the brainstem. In spite of this challenge, recent studies have shown that it is possible to capture functional BOLD activity at the LC region, using high-resolution BOLD scans and neuromelanin-sensitive anatomical scans for the localization of the LC region (Clewett et al., 2018; Hall et al., 2024; Idrees, 2023; Krebs et al., 2013, 2018; Murphy et al., 2014).

In the current study, we set out to investigate for the first time the LC hypothesis of state regulation deficits in ADHD, by assessing LC activity via fMRI, in a sample of adult participants with and without ADHD. Similarly to Metin et al. (2015), we implemented a target detection task with target (Go) and standard (No-Go) trials, presented in three blocks with different event rate levels (fast, moderate, slow). To capture LC activity in the absence of task events, we inserted 20-s resting intervals into the paradigm within each event rate condition. Based on the assumption that LC activity during such resting intervals indexes the tonic aspect of arousal (activation) as conceptualized in the state regulation deficit account and the Cognitive-Energetic Model, we expected resting-interval LC activity to decrease (and reaction time to slow down) as event rate decreases. We further expected this effect to be stronger in adults with ADHD. Note that other researchers have hypothesized an overall higher tonic LC activity in ADHD, based on the notion that stimulant medication acts to suppress tonic LC activity in rats (Mefford & Potter, 1989; Pliszka et al., 1996), or, contrarily, overall lower tonic LC activity in ADHD due to a more general proneness to tonic hypoarousal (Howells et al., 2012). This could also be tested with the data from the present study, where an overall higher resting-interval LC activity in the ADHD group would be expected under the former hypothesis and an overall lower resting-interval LC activity in ADHD under the latter. Regarding event-related LC activity (i.e., in response to target trials), we expected a U-shaped effect across event rate levels, although our prediction was unspecific about the direction of the effect (i.e., regular U or inverted U). On the one hand, following the adaptive gain theory of LC activity by Aston-Jones and Cohen (2005), we would expect event-related LC activity to be optimal during the moderate event rate, which the theory denotes as “phasic mode of LC firing”. In this case, we would expect decreased event-related LC activity during the fast and slow event rate, in other words an inverted U, and this quadratic effect to be stronger in ADHD. On the other hand, there is a large body of literature linking phasic LC activity to cognitive effort (Alnaes et al., 2014; Bornert & Bouret, 2021). According to this notion, in combination with the prediction of the state regulation deficit account of a need for effort allocation during fast and slow event rate to regulate activation levels in order to optimize performance, event-related LC activity may potentially follow a regular-U function, with increased event-related responses during extreme event rates, but less so in ADHD.

In addition to the experimental data, we collected self-reports of state regulation deficits in daily life from all participants by means of a questionnaire, to assess state regulation outside the lab in more natural environments, allowing for a comparison with the experimental indices.

## 2 Method

This study was approved by the Ethics Committee of Ghent University Hospital (approval no. BC-09832) and conducted in accordance with the Declaration of Helsinki and its amendments, as well as Good Clinical Practice standards. Participants received detailed written and verbal information about the aim and the procedure of the experiment. They provided written informed consent prior to their enrolment in the study.

### 2.1 Participants and prescreening

Participants were recruited through social media and word of mouth and selected based on a set of in- and exclusion criteria (see below). The final sample consisted of 27 participants with a formal clinical diagnosis of ADHD (14 male and 13 female, age range 18–40, *M*_age_ = 27.19, *SD*_age_ = 5.51) and 28 control participants (13 male and 15 female, age range 20–43, *M*_age_ = 26.32, *SD*_age_ = 5.78). The groups did not differ in terms of sex ratio, age, and intelligence (see Table 1). Participants were excluded from the ADHD group if they had any co-occurring psychiatric diagnosis, with the exception of learning disorders (i.e., dyslexia or dyscalculia), or if they currently used atomoxetine medication. Control participants were excluded if they had any formal psychiatric diagnosis. Within the ADHD group, presentation subtypes were specified as follows: fourteen participants had a predominantly inattentive presentation, two had the predominantly hyperactive/impulsive presentation, and eight the combined presentation (the remaining three subjects did not provide this information). Eleven participants in the ADHD group had received their diagnosis during childhood or adolescence, and the remaining 16 had received it during adulthood. Participants received a monetary compensation of EUR 50 (ADHD group) or EUR 35 (control group). The difference in compensation was due to the unequal time investment that was required for participation in either group.

**Table 1.** Demographic data, questionnaire scores, and between-group statistical comparisons.

Variable	ADHD group (*n* = 27)	Control group (*n* = 28)	Between-group statistics	
	*M (SD)*	*M (SD)*	Independent *t*-test	Bayesian *t*-test
Age	27.18 (5.51)	26.32 (5.78)	*t*(53) = 0.57, *p* = .573	BF_10_ = 0.31
IQ	104.44 (10.24)	102.64 (9.83)	*t*(53) = 0.67, *p* = .501	BF_10_ = 0.37
6-q ASRS sum score	17.81 (3.03)	8.21 (2.77)	*t*(53) = 12.29, *p* < .001***	BF_10_ > 1000
18-q ASRS sum score	49.22 (8.78)	23.86 (7.52)	*t*(53) = 11.53, *p* < .001***	BF_10_ > 1000
SRS-Total	56.62 (19.02)	35.07 (20.80)	*t*(53) = 4.01, *p* < .001***	BF_10_ > 1000

	Median (min-max)	Median (min-max)	Mann-Whitney *U-*test	Bayesian M-W test
ASR-ADHD	77 (61 - 93)	53 (50 - 69)	*U* = 17.0, *p* < .001***	W BF_10_ > 1000
ASR-depression	66 (50 - 83)	54 (50 - 92)	*U* = 230.0, *p* = .006**	W BF_10_ = 1.89
ASR-anxiety	52 (50 - 73)	51.5 (50 - 70)	*U* = 292.5, *p* = .073	W BF_10_ = 0.62
ASR-substance abuse	56 (50 - 64)	55 (50 - 66)	*U* = 385.5, *p* = .374	W BF_10_ = 0.28

*Note.* Male-to-female ratio was 14:13 in the ADHD group and 13:15 in the control group. A chi-square test indicated no group difference in sex ratio, X^2^ = 0.16, *p* = .688, BF_10_ = 0.35. Handedness ratio right:left was 21:6 in the ADHD group and 28:0 in the control group. Most participants reported that they currently had at least a part-time occupation (ADHD = 26 and control = 25). All subjects had at least completed secondary education tracks and a large part of the participants had completed science-related higher education tracks (ADHD = 12 and control = 19). Moreover, many participants reported that they were currently following higher education programs (ADHD = 16 and control = 23). *ASRS*: ADHD Self-Report Scale. *SRS-Total*: Total score of the Social Responsiveness Scale. *ASR*: Achenbach Adult Self-Report questionnaire. All four ASR scales are from the DSM-section of the ASR. Bayes Factor BF_10_ represents the likelihood ratio of the alternative hypothesis vs the null-hypothesis for additionally calculated Bayesian tests. W BF: Bayesian Mann-Whitney Test BF, obtained through 5 chains of 1000 iterations (convergence statistic $\hat{R}$ ranged between 1.000 and 1.003 for the four tests reported here).

** *p* < .01, *** *p* < .001.

All (potential) participants filled in online versions of a series of questionnaires. Firstly, to assess ADHD symptoms, the Adult ADHD Self-Report Scale v1.1 (ASRS) was used, which has a high validity and reliability (Adler et al., 2006; Kessler et al., 2005). It comprises 18 questions that probe for symptoms of ADHD, one example being “How often do you have trouble wrapping up the final details of a project, once the challenging parts have been done?”. Responses are collected on a 5-point Likert scale ranging from “never” to “very often”. Given the separate literature on the short (6-question) screener version of the ASRS, which consists of the first six questions, and on the full (18-question) version, we calculated the sum scores for both versions. Secondly, participants filled in the Dutch adult self-report version of the Social Responsiveness Scale (SRS; Constantino & Gruber, 2005; Roeyers et al., 2011), to screen out cases of elevated autism symptomatology in both participant groups (total scale score, with a clinical cutoff of 75). To collect demographic data and to assess the influence of other common co-morbid symptom clusters, participants also filled in the Achenbach Adult Self-Report screening questionnaire (ASR; Achenbach & Rescorla, 2003). On the ASR, the relevant scales were the DSM scales for ADHD, for depression, for anxiety, and for substance abuse, which all have a clinical cutoff value of 70. Finally, we collected self-reports of handedness using the Edinburgh Handedness Inventory (Oldfield, 1971). Participants were excluded from the ADHD group if they obtained a clinical score on the SRS questionnaire indicating elevated symptoms of autism spectrum disorder, or a clinical score on the substance abuse scale of the ASR. Control participants were excluded if they had any formal psychiatric diagnosis, or if they scored in the clinical range on the SRS, the ASRS, the ADHD scale of the ASR, or the substance abuse scale of the ASR.

For all participants in the ADHD group, the diagnosis was verified using the Dutch version of the DIVA-5, a semi-structured diagnostic interview based on the DSM-5 criteria (Hong et al., 2020; Kooij et al., 2019; Zamani et al., 2021). The interviews were conducted through video conferencing by a board-registered clinical psychologist and took approximately 90 min to complete. Only participants who met the diagnostic criteria for ADHD on the DIVA-5 were included in the experiment.

In addition to the questionnaires, cognitive ability was assessed for all participants. More specifically, we obtained scaled scores of the subtests Matrix Reasoning and Vocabulary from the Wechsler Adult Intelligence Scale, Dutch Version (WAIS-IV-NL; Wechsler, 2012). Based on these scaled scores, we then estimated the two-subtest full-scale intelligence quotient (FSIQ-2) of the Wechsler Abbreviated Scale of Intelligence, Second Edition (WASI-II; McCrimmon & Smith, 2013; Wechsler, 2011). An IQ estimate below 80 was set as an exclusion criterium.

A subset of the included participants in the ADHD-group (*n* = 15) was using stimulant medication at the time of the experiment and were requested to refrain from using the stimulant medication during the 48 h preceding the MRI session and cognitive testing. Participants were not asked to interrupt the use of any other medication. Three subjects reported using antidepressants during the course of the experiment.

As an experimental measure rather than a prescreening tool, participants also filled in the State Regulation Deficit Questionnaire (SRDQ), a questionnaire developed by members of our research lab, which can be found in Drescher et al. (2021). This questionnaire probes for the frequency of state regulation deficits in daily life through 10 questions that use both examples of overstimulation and understimulation, an exemplary item being “I have difficulty concentrating when a task has little variation”. Responses are collected on a 5-point Likert scale ranging from “much less than average” to “much more than average”. Importantly, the SRDQ addresses the lack of research into the ecological validity of state regulation deficits. Previous research using this questionnaire found that state regulation deficit reports in daily life tend to converge with behavioral performance deficits (Drescher et al., 2021, 2023).

### 2.2 Task design and behavioral analysis

The task was built in PsychoPy v2021.2.2 (Peirce et al., 2019) and carefully piloted prior to its implementation. It consisted of a target detection paradigm with similar specifications as in previous research (Drescher et al., 2021, 2023; Metin et al., 2015). A black fixation cross was shown in the center of a gray screen and intermittently replaced by either the infrequent target trials (the letter “Q”, 30% of the trials) or the frequent standard trials (the letter “O”, 70% of the trials). Both letters were also shown in black. Three event rate conditions were presented in counterbalanced order: a fast event rate condition (average stimulus-onset asynchrony = 2.0 s), a moderate event rate condition (average stimulus-onset asynchrony = 4.0 s), and a slow event rate condition (average stimulus-onset asynchrony = 8.0 s). Importantly, the stimulus-onset asynchrony was jittered such that trials had a varying duration, namely 1.0–4.0 s in the fast event rate, 3.0–6.0 s in the moderate event rate, and 7.0–10.0 s in the slow event rate. The intervals were drawn from exponential distributions with the abovementioned averages and boundaries. The order of the trials was quasi-randomized by shuffling quintuplets of trials that were pre-arranged to ensure that targets were not frequently followed by another target. Trial order and trial durations were not altered between participants.

Participants were instructed to respond by pressing a button on the response box with the index finger of their dominant hand, with equal emphasis on speed and accuracy. Before carrying out the task, they completed 10 practice trials with feedback about the accuracy of their response. A 20-s resting interval was inserted at the beginning of each event rate condition as a reference, and at the middle of the condition (i.e., after exactly half of the trials) to measure the impact of the condition on LC activity. Between conditions, participants were given a short break (30 s) during which they were instructed to let their eyes rest.

Behavioral performance was analyzed by studying the effects of event rate and ADHD status on reaction time and reaction time variability. To this end, we calculated mixed ANOVA models with the respective behavioral measure as dependent variables. Since normality testing with a Shapiro-Wilk test indicated that the majority of individual reaction time distributions per participant and condition (100 out of 165) significantly differed from a normal distribution, we used the median as the central tendency measure of individual reaction time values, as well as a quartile-based measure for individual reaction time variability values (inter-quartile range / median). On the group level, these median-based reaction time and reaction time variability values were generally normally distributed (10 out of 12 bins), which allowed the use of frequentist ANOVA models. Bayesian models were also calculated next to the frequentist instantiations, and the Bayes’ Factor (BF) is reported for each effect hypothesis, wherever its calculation was possible. For the statistical analysis, we used both SPSS v28 and JASP (v0.17.3; https://jasp-stats.org/). Error rates were not analyzed, as accuracy on the task was very high, with a substantial subset of the participants committing no errors at all and thereby severely reducing power for statistical analysis.

### 2.3 MRI data acquisition and analysis

The Magnetic Resonance Imaging (MRI) scans were acquired on a Siemens Magnetom Prisma 3.0 Tesla MRI system (Siemens Medical Systems; Erlangen, Germany) with a 64-channel head coil. First, an anatomical T1-weighted MPRAGE scan was obtained, with GRAPPA mode (acceleration = 2), repetition time (TR) = 2250 ms, echo time (TE) = 4.18 ms, inversion time (TI) = 900 ms, Field of View (FoV) = 256 mm, Flip angle (FA) = 9°, and voxel size = 1.0*1.0*1.0 mm. For the localization of the LC within the brainstem, the protocol furthermore included a T1-weighted anatomical Turbo-Spin-Echo (TSE) sequence, which is susceptible to the neuromelanin pigmentation of the LC neurons, TR = 559 ms, TE = 9.8 ms, FoV = 192 mm, FA1 = 70°, FA2 = 180°, 10 interleaved slices, and voxel size 0.5*0.5*3.0 mm. The TSE scan was oriented perpendicular to the brain stem and covered the section of the pons. During the target detection task, T2*-weighted functional BOLD images were acquired, with multiband acceleration factor 3, TR = 2090 ms, TE = 27.0 ms, FoV = 192 mm, FA = 79°, 69 interleaved slices, 885 volumes, and voxel size 1.714*1.714*2.0 mm. Moreover, for the correction of field magnetization inhomogeneities, a GRE field map was obtained, TR = 672 ms, TE_1_ = 4.92 ms, TE_2_ = 7.38 ms, FoV = 192 mm, FA = 90°, 69 slices, and voxel size = 3.0*3.0*2.0 mm.

Note that the protocol of this experiment also included a separate functional run during rest and an additional T2-weighted anatomical scan. These scans are part of another study.

The preprocessing of the anatomical T1-weighted scan and the functional BOLD series was carried out in fMRIPrep v21.0.1 (Esteban et al., 2019). An automated description of the exact methodology within the preprocessing pipeline can be found in the Supplementary Materials (Section A). Importantly, the BOLD series was head-motion corrected, field-map (susceptibility distortion) corrected, and co-registered to the T1-weighted anatomical reference. The preprocessed BOLD series in native space (output derivative ‘desc-preproc_bold’) were used for the subsequent processing steps. To demonstrate that the distortion correction was successful, we provide visual comparisons of distorted and corrected images of the brainstem region in the Supplementary Materials (Section B). Since possible group differences in motion parameters are a potential confound for the results of the fMRI data, we moreover compared the mean standardized DVARS (Data VARiance Statistic) and the mean framewise displacement output from fMRIPrep across groups. Groups were equal, both for standardized DVARS, ADHD *M* = 1.202 (*SD* = 0.051), Control *M* = 1.221 (*SD* = 0.065), *t*(53) = 1.238, *p* = .221, *d* = .334, and for framewise displacement, ADHD *M* = 0.177 (*SD* = 0.062), Control *M* = 0.151 (*SD* = 0.098), *t*(53) = 1.19, *p* = .239, *d* = .321.

**Fig. 1.**
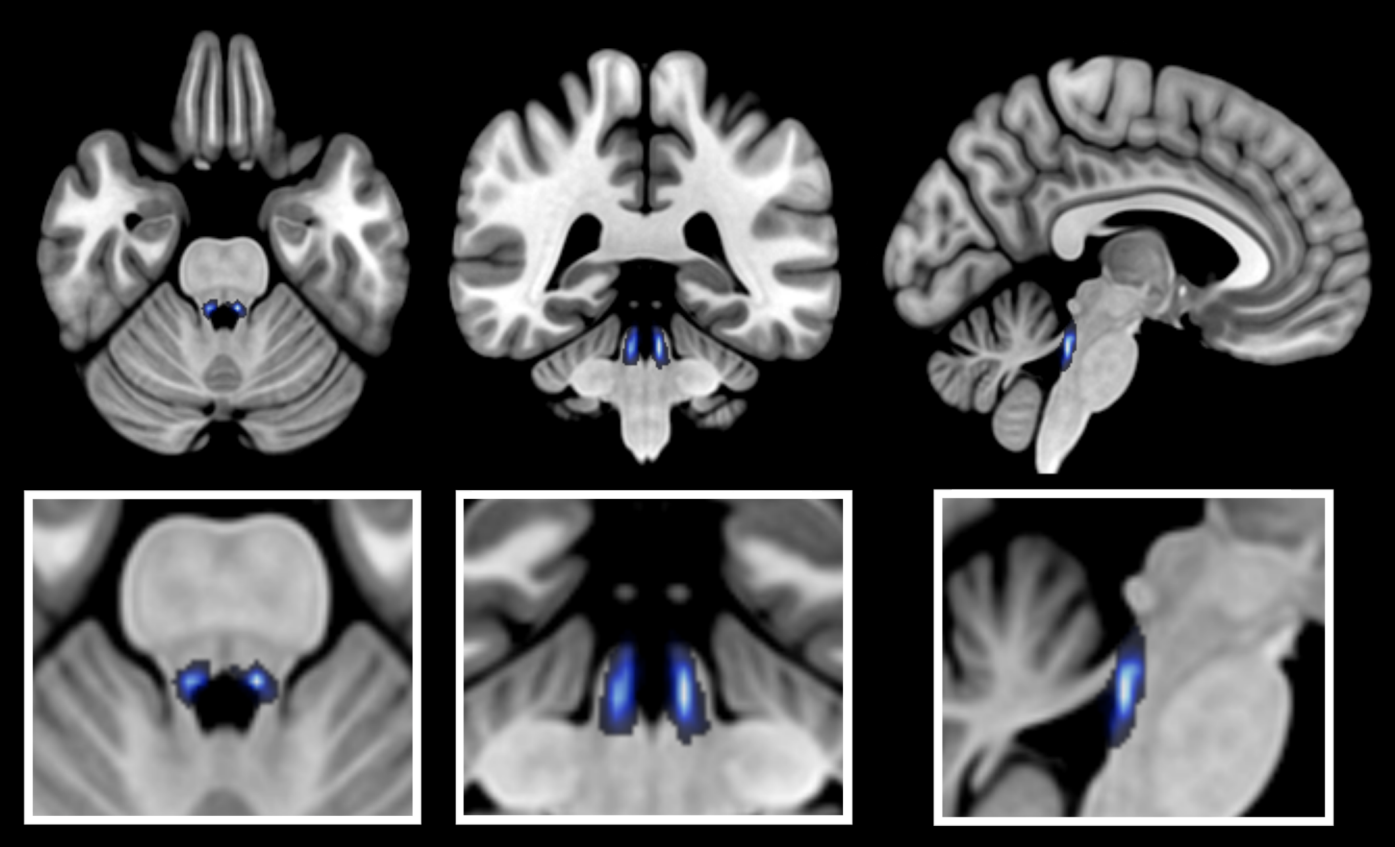
Anatomical location of the Locus Coeruleus masks in normalized space (axial, coronal, and sagittal view). The image shows the individual masks combined into an overlay heatmap and projected onto the SPM152 brain template.

To define the individual LC region of interest (ROI) for each participant, the raw TSE scans were first co-registered to the native space, using the toolkit Statistical Parametrics Mapping v12 (SPM12; https://www.fil.ion.ucl.ac.uk/spm/software/spm12/). More specifically, the raw TSE scans were co-registered to the respective T1-weighted anatomical references, thereby reslicing the TSE scans to a resolution of 1.0*1.0*1.0 mm. Next, three-dimensional ROI masks of the LC were manually tagged for each participant using the software MRIcroGL (https://www.nitrc.org/projects/mricrogl/; Rorden & Brett, 2000). These masks were drawn based on the visible contrast in the neuromelanin-sensitive TSE scan, at the typical anatomical location of the LC. The mask locations in native space are visualized in the Supplementary Materials (Section C). Subsequently, the TSE scans, the LC masks, and the preprocessed BOLD series in native space were all normalized to the default brain template of SPM12. Within this processing step, the TSE scan and the ROI masks were moreover resliced to the resolution of the BOLD series (1.714*1.714*2.0 mm). No smoothing was applied to the images at any point of the processing. To confirm that the normalizing procedure resulted in an optimal alignment between the scans and the SPM template, the output was subjected to systematic visual overlay comparison. Furthermore, the position of the (normalized and resliced) individual LC masks was verified based on the LC contrast of the normalized and resliced TSE scans, and the masks were slightly corrected to optimize their overlap with the respective LC contrast in normalized space. The coordinates of the combined LC masks across all subjects ranged from 2 mm to 8 mm (right LC) and from -7 mm to -1 mm (left LC) along the lateral (x) axis, from 34 mm to 42 mm along the anterior-posterior (y) axis, and from -15 mm to -38 mm along the inferior-superior (z) axis (coordinates obtained in MRIcroGL). Figure 1 visualizes the location of the combined masks in normalized space.

To assess data quality of the functional signal within the LC ROI, we obtained the temporal signal-to-noise-ratio (tSNR) within the LC mask by computing the mean value over time divided by the standard deviation, averaged across all voxels of the LC mask. The average tSNR across all subjects was *M* = 30.55 (*SD* = 6.06), which is above the standard cutoff of < 30 (Grueschow et al., 2021; He et al., 2023).

For the analysis of the BOLD activity within the ROI of the LC, first-level regression models were computed in SPM12 for each participant, based on the preprocessed and normalized BOLD time series. These regression models included twelve condition-specific regressors, which were convolved with the default canonical hemodynamic response function: the resting intervals of each event rate condition (pre-condition and mid-condition resting interval) to obtain coefficients of tonic LC activity, and the target and standard trials of each event rate condition to model the event-related LC activity. A thirteenth regressor was included for all time periods that would not be part of the further analysis, including incorrect trials and breaks. The toolkit MarsBaR 0.45 (https://marsbar-toolbox.github.io/; Brett et al., 2002) was subsequently used to extract the beta-values of BOLD activity ROI from the model, at the LC location from each participant’s regression model, using the individual ROI masks in normalized space.

The beta values of LC activity were then subjected to the second-level statistical analysis. We opted to compute separate ANOVA models, one for the tonic LC data (i.e., the LC values obtained during the 20-s resting intervals) and one for the event-related LC data (i.e., the LC values during the task trials). In each ANOVA model, we compared the type of event (within-subjects), the groups (between-subjects), and the event rate condition (within-subjects), in a 2 × 2 × 3 ANOVAs. The type of event factor of the resting-interval LC ANOVA model was coded as the resting interval at the start vs. at the midpoint of the block. In the event-related LC ANOVA, this factor was defined as target trials vs. standard trials.

As an exploratory analysis step, we carried out correlational analyses to further assess the assumption of a relationship between LC activity, behavior, and state regulation. To quantify individual regulatory effort in event-related LC activity, we calculated the difference in event-related LC values between the slow and the fast event rate condition and correlated it with the corresponding performance decrement (i.e., the difference in reaction time between both conditions), analogous to the approach in previous pupillometry research (Drescher et al., 2023). Moreover, we tested the correlation of the event-related LC difference with SRDQ scores. To quantify overall resting-interval activity, we averaged values across all resting intervals per participant. Correlations were then computed between average resting-interval LC and the performance decrement between the fast and the slow event rate, as well as with the SRDQ scores.

Note that throughout the experiment, we also recorded pupil size in the scanner (as a proxy of LC activity; Aston-Jones & Cohen, 2005; Elman et al., 2017; Joshi et al., 2016; Murphy et al., 2014). However, due to technical issues, a considerable portion of the data was lost or of insufficient quality, precluding the further analysis of these recordings.

## 3 Results

### 3.1 Questionnaire results

The SRDQ indicated that the ADHD group reported significantly more state regulation deficits in their daily life (*M* = 29.67, *SD* = 3.80) than the control group (*M* = 19.04, *SD* = 5.07), *t*(53) = 8.78, *p* < .001, Cohen’s *d* = 2.37, BF_10_ > 1000. In addition, the state regulation deficit sum scores were positively correlated with the dimensional short-version ASRS scores, indicating that symptoms of ADHD are also dimensionally related to state regulation deficits in daily life, Pearson’s correlation coefficient *r*(53) = 0.798, *p* < .001, BF_10_ > 1000. The internal consistency was acceptable for the SRDQ (Cronbach’s α = 0.611), although somewhat lower than in previous experiments that applied the dimensional approach to ADHD (Drescher et al., 2021, 2023).

### 3.2 Behavioral results

The number of omission and commission errors was very low, which is unsurprising given the choice of the task. Errors were, therefore, not further analyzed. Reaction time increased linearly with decreasing event rate, replicating prior basic findings from event rate research (i.e., slowest responses during the slowest event rate; see, for example, Epstein et al., 2011; Kooistra et al., 2010; Metin et al., 2015; Wiersema, van der Meere, Antrop, et al., 2006). None of the other main effects or interaction effects were significant (see Table 2 for descriptive statistics and mixed ANOVA results).

**Table 2.** Mixed ANOVA models for the behavioral performance measures.

Effect		Median reaction time	Reaction time variability^a^
Condition (event rate)	F-value	*F*(2,106) = 31.928	*F*(1.9,98.1) = 1.942
	Significance	*p* < .001***	*p* = .152
	Effect size	η^2^_p_ = .376	η^2^_p_ = .035
	Bayes’ factor	BF_10_ > 1000	BF_10_ = 0.234
Group	F-value	*F*(1,53) = 0.382	*F*(1,53) = 0.269
	Significance	*p* = .539	*p* = .606
	Effect size	η^2^_p_ = .007	η^2^_p_ = .005
	Bayes’ factor	BF_10_ = 0.314	BF_10_ = 0.212
Condition × Group Interaction	Type	Linear contrast	Quadratic contrast
	F-value	*F*(1,53) = 0.10	*F*(1,53) = 0.49
	Significance	*p* = .759	*p* = .486
	Effect size	η^2^_p_ = .002	η^2^_p_ = .009

a Huynh-Feldt correction was applied, since Mauchly’s test indicated that the sphericity assumption was not met. BF_10_ indicates the Bayesian likelihood estimate for the alternative hypothesis, relative to the null hypothesis, for the additionally calculated Bayesian model.

*** *p* < .001.

### 3.3 fMRI results

**Fig. 2.**
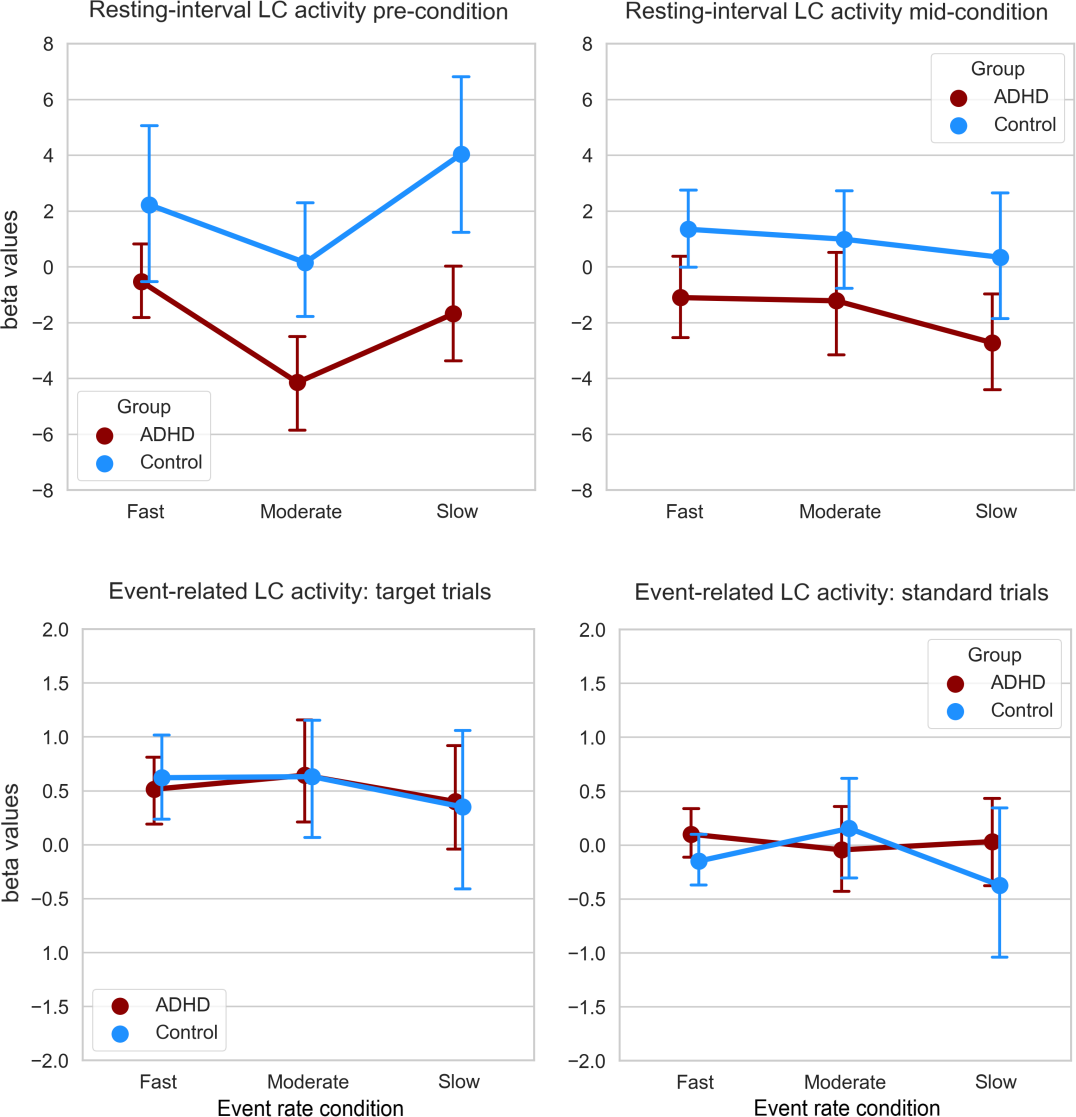
Resting-interval (above) and event-related (below) LC activity data. Graphs are split for the Type of event (within-subjects) factor. Error bars represent ±1 standard error around the mean.

The results of the resting-interval and the event-related 2 × 2 × 3 ANOVA models for the beta values of LC activity are listed in Table 3 and graphically represented in Figure 2. A significant main effect of Group was found for the overall resting-interval LC activity, indicating that LC activity was generally lower in the ADHD group, irrespective of condition or the type of resting interval (pre- or mid-condition). Additionally, we found that event-related LC activity was significantly higher during target trials compared to standard trials, irrespective of Group or Condition. The remaining effects, including all the interactions, were non-significant.

**Table 3.** Results of mixed ANOVA models for resting-interval and event-related Locus Coeruleus activity.

Effect		Resting-interval LC activity	Event-related LC activity
	Comparison	Pre- vs mid-condition resting interval	Standard vs target trial
Type of event	F-value	*F*(1, 53) = 0.116	*F*(1, 53) = 6.314
	Significance	*p* = .735	*p* = .015*
	Effect size	η^2^_p_ = .002	η^2^_p_ = .106
	Bayes’ factor	BF_10_ = 0.085	BF_10_ = 0.694
Condition (event rate)	F-value	*F*(2.0, 108.2) = 0.594^a^	*F*(1.9, 98.7) = 0.172^a^
	Significance	*p* = .554	*p* = .827
	Effect size	η^2^_p_ = .011	η^2^_p_ = .003
	Bayes’ Factor	BF_10_ = 0.032	BF_10_ = 0.040
Group	F-value	*F*(1, 53) = 7.017	*F*(1, 53) = 0.039
	Significance	*p* = .011*	*p* = .843
	Effect size	η^2^_p_ = .117	η^2^_p_ < .001
	Bayes’ Factor	BF_10_ = 0.944	BF_10_ = 0.092
Type of event × Group	F-value	*F*(1, 53) = 0.506	*F*(1, 53) = 0.132
	Significance	*p* = .480	*p* = .717
	Effect size	η^2^_p_ = .009	η^2^_p_ = .002
	Bayes’ Factor	BF_10_ = 0.059	BF_10_ = 0.055
Type of event × Condition (event rate)	F-value	*F*(1.8, 94.4) = 1.329^a^	*F*(1.9, 99.6) = 0.005^a^
	Significance	*p* = .268	*p* = .993
	Effect size	η^2^_p_ = .024	η^2^_p_ < .001
	Bayes’ factor	BF_10_ = 0.007	BF_10_ = 0.008
Group × Condition (event rate)	F-value	*F*(2, 106) = 0.197	*F*(1.9, 98.7) = 0.071^a^
	Significance	*p* = .821	*p* = .921
	Effect size	η^2^_p_ = .004	η^2^_p_ = .001
	Bayes’ factor	BF_10_ = 0.012	BF_10_ = 0.006
Type of event × Group × Conditon (event rate)	F-value	*F*(1.8, 94.4) = 0.108^a^	*F*(1.9, 99.6) = 0.221^a^
	Significance	*p* = .876	*p* = .788
	Effect size	η^2^_p_ = .002	η^2^_p_ = .004
	Bayes’ factor	BF_10_ = < 0.001	BF_10_ < 0.001

a Huynh-Feldt correction was applied, since Mauchly’s test indicated that the sphericity assumption was not met. BF_10_ indicates the Bayesian likelihood estimate for the alternative hypothesis, relative to the null hypothesis, for the additionally calculated Bayesian model. Resting-interval LC activity and Event-related LC activity are the beta values for the Locus Coeruleus region-of-interest, derived from a multiple regression model of the whole-brain functional activity, with regressors being resting intervals or task trials, respectively.

* *p* < .05.

To specifically test the hypothesis of a decline in resting-interval LC activity with decreasing event rates, and a stronger decline in the ADHD group, we computed linear contrasts on the mid-condition interval alone. The linear contrast of the event rate main effect was not significant, *F*(1, 53) = .478, *p* = .492, η^2^_p_ = .009, and neither was the linear Event Rate × Group interaction contrast, *F*(1, 53) = .026, *p* = .872, η^2^_p_ < .001. To also test the hypothesis of a U-shaped effect on event-related LC activity, we computed quadratic contrasts specifically on the target trials, which was not significant for the main effect of event rate, *F*(1, 53) = .131, *p* = .719, η^2^_p_ = .002, nor for the Event Rate × Group interaction, *F*(1, 53) = .002, *p* = .965, η^2^_p_ < .001.

To confirm that the main effect of trialtype effectively represents a difference in activity at the LC location, we carried out a post-hoc voxel-wise contrast analysis, which, indeed, showed a spatial correspondence of the contrast activation and the LC ROI. This analysis and its results can be found in the Supplementary Materials (Section D).

### 3.4 Exploratory correlational analyses

The correlation between the event-related LC activity difference (fast-minus-slow event rate) and the performance (reaction time) decrement between the fast and the slow event rate was not significant, *r* = -.028, *p* = .837, BF_10_ = 0.172, and neither was the correlation between the event-related LC activity difference and SRDQ scores, *r* = .094, *p* = .497, BF_10_ = 0.211. The average resting-interval LC activity was not significantly correlated with the reaction time difference, *r* = -.021, *p* = .878, BF_10_ = 0.170. However, average resting-interval LC activity was significantly negatively associated with SRDQ scores, *r* = -.351, *p* = .009, BF_10_ = 4.851. This last finding appeared to be driven by group differences, however. When partialling out the effect of Group, the correlation between average resting-interval LC activity and SRDQ scores was not significant, *r* = -.146, *p* = .291.

## 4 Discussion

In the present study, we investigated the hypothesized involvement of the LC in state regulation deficits in ADHD. We collected high-resolution fMRI data to assess LC activity in adults with and without ADHD, while they carried out a target detection task at three event rates.

We found no evidence of a decline in resting-interval LC activity (at the mid-condition interval) with decreasing event rates, contrary to our hypothesis. This is surprising, as participants did exhibit slower reactions with decreasing event rates, in line with previous event rate studies, suggesting that the event rate manipulation was effectively modulating activation. We moreover found no interaction of Event Rate by Group for resting-interval LC activity, confirmed by the non-significant linear contrast run specifically on the mid-condition resting interval. It could be that in our current study, the event rate manipulation did not (sufficiently) impact the activation level, yet this does not seem likely taking into account the reaction time findings. Alternatively, the findings may imply that resting-interval LC activity as measured in our study is not a suitable measure of the activation pool as conceptualized in the state regulation deficit account. The Cognitive-Energetic Model (and by extension, the state regulation deficit account) presumes the classic information processing stream from stimulus to response, and the tonic activation pool in this framework is related to motor readiness, mediated mainly by dopaminergic activity (Pribram & McGuinness, 1975; Riedel et al., 2006; Sanders, 1983). As part of a different line of literature, the LC/adaptive gain theory by Aston-Jones and Cohen (2005) describes a more general tonic arousal state, primarily related to noradrenergic activity. There is considerable overlap between the LC/adaptive gain theory and the Cognitive-Energetic Model/state regulation deficit account, as both comprise an inverted U-curve describing the relationship between tonic arousal and performance. This overlap suggests that LC-NE functioning could be implicated in state regulation deficits in ADHD (Bellato et al., 2020; Howells et al., 2010; Imeraj et al., 2012; Konrad et al., 2006; Rowe et al., 2005; Sonuga-Barke et al., 2010; van der Meere, 2005). However, our current results may imply that activation as referred to in the state regulation deficit account and argued to be implicated in ADHD, is a separate process not (directly) related to LC activity. This warrants further research into how the state regulation deficit and LC theories relate.

In spite of the absence of an event rate effect on resting-interval LC activity, we found that it was overall lower in the ADHD group, in line with the hypothesis of a general tonic LC hypo-arousal in ADHD (Howells et al., 2012). Indeed, a series of theoretical approaches to ADHD presume a general proneness to (tonic) under-arousal in ADHD, for which the symptoms (and stimulant medication) appear to have a compensatory function. Importantly, the present result suggests an implication of the LC-NE system in this general hypo-arousal. This corresponds to the high efficacy of atomoxtine medication in ADHD, which is a noradrenergic agonist. Our results are, therefore, in contrast with the contrary hypothesis of a general tonic LC “overdrive” in ADHD (Mefford & Potter, 1989; Pliszka et al., 1996). It should be noted here, however, that the idea of a tonic LC overdrive in ADHD was based on research in rats, and was never tested in humans with ADHD.

Event-related LC activity was found to be stronger during target trials than standard trials. This is consistent with previous evidence indicating sensitivity of the LC to behaviorally relevant and/or deviant stimuli (Aston-Jones & Cohen, 2005), and it is in line with previous fMRI research analyzing LC activity during a target detection task (Krebs et al., 2018; Murphy et al., 2014). The current result, therefore, corroborates that our ROI analysis succeeded in assessing LC activity, in addition to the aforementioned main group difference in resting-interval LC activity, although both results were not corrected for multiple testing and should be interpreted with caution. However, against our predictions, no other significant effects were found for event-related LC activity; we observed no main effect of Event Rate, no main Group effect, and no Event Rate by Group interaction effect. In addition, the quadratic contrasts specifically assessing U-shaped effects were non-significant. These findings disconfirm our hypothesis of a U-shaped event rate effect on event-related LC activity. Exploratory correlational analyses also did not show an association between changes in event-related LC activity induced by the event rate manipulation, and corresponding performance changes, nor were changes in event-related LC activity associated with self-reported state regulation difficulties in daily life. It could be argued that this is due to the fact that we did not observe group differences in performance in our study. Within this respect, it is important to mention that previous event rate studies in an fMRI context also failed to find Group by Event Rate interaction effects for performance, although they did in fact find Group by Event Rate interactions for activity in other brain regions (Kooistra et al., 2010; Metin et al., 2015). The absence of a behavioral interaction could be due to our paradigm that was optimized for fMRI research, using a target detection task instead of the commonly used Go/NoGo task in previous behavioral event rate studies in ADHD, which may be less sensitive to detect state regulation difficulties at the behavioral level (Metin et al., 2015). While this is a possible explanation for the absence of group differences at the behavioral level, it is unlikely that this explains the absence of Group by Event Rate interactions for LC activity. Our findings rather point to fact that the LC does not play the hypothesized role in state regulation deficits in ADHD, and that other brain regions may be implicated instead.

One of the previous fMRI studies that investigated event rate effects on the activity of the Default Mode Network (DMN) in ADHD found that adults without ADHD showed more supression of DMN activity during the fast and slow than during the moderate event rate condition, while this effect was absent in adults with ADHD (Metin et al., 2015). In other words, attenuation of DMN activity was found to be disrupted in adults with ADHD during fast and slow event rate levels, but not during the moderate event rate level. The DMN comprises a set of brain regions whose activity is increased during rest (or low task-demand) and attenuated when task-related attentional demands increase (Raichle & Snyder, 2007). Based on this, the authors reasoned that the ADHD-related deficit in regulating activation through effort allocation may be mediated by unattenuated DMN activity (Metin et al., 2015). They further hypothesized that the LC may play an important role herein, as noradrenergic projections from the LC could be important in fine-tuning the balance between task-postive networks and task-negative networks (i.e., the DMN). Previous research has, indeed, found that this balance is disrupted in ADHD (Sidlauskaite et al., 2016a, 2016b). However, the findings from our current study, in which we applied a similar paradigm, do not provide support for the hypothesis of an involvement of the LC-NE system in such context-dependent network activations and performance adaptations, and by extension in the momentary deficits of state regulation in ADHD. Rather, we found evidence for overall lower resting-interval LC activity in ADHD irrespective of event rate condition, which suggests a general proneness to a tonic LC hypoarousal (Howells et al., 2012). This calls for further investigation into the link between this finding and (neurobiological underpinnings of) state regulation deficits in ADHD.

It is noteworthy that we found that adults with ADHD included in our study reported more state regulation deficits in their daily life, as assessed with the SRDQ. This result is in line with the findings from two previous studies that showed more self-reported state regulation deficits in adults with elevated ADHD traits (Drescher et al., 2021, 2023).

The present study has certain limitations. As mentioned above, the paradigm may not have been optimal to investigate state regulation difficulties at the performance level, as it was optimized for fMRI rather than for the replication of prior behavioral results. In short, the task is an easy attentional oddball task with infrequent Go-trials and frequent No-Go trials (in contrast to a Go/No-Go task as used in many other studies). As such, this task is intended to produce a low error rate, thereby allowing for a maximum statistical power when analyzing the neurobiological data of the trials with correct responses (Metin et al., 2015). This design consequently leads to constraints, especially for error rate analysis, but it may simultaneously lead to lower sensitivity for other (e.g., state regulation deficit-related) parameters. However, one of the previous fMRI studies with ADHD vs. control participants used a Go/No-Go task with two event rate levels, and found no Event Rate by Group interaction effect either (Kooistra et al., 2010). An alternative (or additional) explanation for the lack of a behavioral interaction effect in these experiments may be that the MRI setting differs from a regular computer lab setting in such a way that it compensates for certain behavioral outcomes. The high level of auditive stimulation may have precluded the expected interaction effects to take place. In addition, the relatively strong jitter on the inter-trial interval used in these three fMRI experiments, which is necessary to avoid predicatibility of the stimulus event and to improve statistical efficiency of the trial-level analysis (Dale, 1999), may have contributed to the absence of an interaction effect. Although speculative, it may also explain the absence of significantly higher reaction time variability in ADHD, as strong jitter has been found to improve reaction time variability specifically in (children with) ADHD (Ryan et al., 2010). An important caveat regarding ROI beta values is that they are referenced to each participant’s (implicit) baseline. The beta values derived from event-related or resting-interval regressors, therefore, represent deviations from these individual baselines and do not allow inferences about absolute LC activity. While this limits their interpretability on an individual level, group-level analyses may still capture systematic differences in relative LC modulation under the assumption that individual baseline offsets are not systematically biased between groups. We acknowledge, however, that this assumption cannot be directly tested with standard BOLD fMRI. Another limitation is related to power. Although the present study involved a larger sample than Metin et al. (2015), and while we did observe group differences for overall resting-interval LC activity, statistical power may have been non-optimal for other effects to be observed, especially in light of the constraints mentioned before. Finally, it could be argued that we did not succeed in reliably capturing LC activity. However, this is unlikely since we followed state-of-the-art procedure including high-resolution TSE scans, high-resolution BOLD scans at 1.7*1.7*2 mm voxel size, field map correction, and various procedural checkpoints to verify data quality. Our methodology is as rigorous and technologically advanced as in previous studies with 3T scanners that have successfully investigated LC activity (Clewett et al., 2018; Hall et al., 2024; Krebs et al., 2013, 2018; Murphy et al., 2014; Payzan-LeNestour et al., 2013). Additionally, we found the expected basic trial effect for event-related LC activity, with greater activity for targets than standards, and we found an overall group effect for resting-interval LC activity.

In spite of these limitations, taken together, our findings cast doubt on the proposed direct involvement of the LC in state regulation deficits in ADHD, as we did not find an effect of the event rate manipulation on resting-interval or event-related LC activity, and no interactions with Group for these measures. Rather, adults with ADHD showed overall lower resting-interval LC activity levels, which could indicate a general proneness to tonic LC-mediated underarousal in ADHD (Howells et al., 2012). As our study was the first to investigate LC BOLD activity (using ROI measures) during an event rate manipulation with an ADHD vs a control group, future research is warranted to replicate our findings and to further investigate the underlying neurobiology of state regulation deficits in ADHD.

## Supplementary Material

Supplementary Material

## Data Availability

Experimental data may be shared upon approval by the Ethics Committee of Ghent University Hospital and the completion of a GDPR-conform data sharing agreement. The paradigm and analysis code is publicly available on GitHub: https://github.com/l-drescher/codes_fMRI_LC_ADHD.
