# Supplementary material for: An fMRI examination of the role of the Locus Coeruleus in state regulation in ADHD
Source: Imaging Neurosci (Camb). 2026 Apr 13;4:IMAG.a.1200. doi: 10.1162/IMAG.a.1200 (PMC13081741; doi:10.1162/IMAG.a.1200)

Supplementary Materials

Section A: FMRIprep methodology

Adapted text output from FMRIprep

Results included in this manuscript come from preprocessing performed using *fMRIPrep* 21.0.1 (Esteban, Markiewicz, et al. (2018); Esteban, Blair, et al. (2018); RRID:SCR_016216), which is based on *Nipype* 1.6.1 (Gorgolewski et al. (2011); Gorgolewski et al. (2018); RRID:SCR_002502).

Preprocessing of B_0 inhomogeneity mappings

A B_0 nonuniformity map (or *fieldmap*) was estimated from the phase-drift map(s) measure with two consecutive GRE (gradient-recalled echo) acquisitions. The corresponding phase-map(s) were phase-unwrapped with `prelude` (FSL 6.0.5.1:57b01774).

Anatomical data preprocessing

The T1-weighted (T1w) image was corrected for intensity non-uniformity (INU) with `N4BiasFieldCorrection` (Tustison et al. 2010), distributed with ANTs 2.3.3 (Avants et al. 2008, RRID:SCR_004757), and used as T1w-reference throughout the workflow. The T1w-reference was then skull-stripped with a *Nipype* implementation of the `antsBrainExtraction.sh` workflow (from ANTs), using OASIS30ANTs as target template.

Functional data preprocessing

For each subject's BOLD run, the following preprocessing was performed. First, a reference volume and its skull-stripped version were generated using a custom methodology of *fMRIPrep*. Head-motion parameters with respect to the BOLD reference (transformation matrices, and six corresponding rotation and translation parameters) were estimated before any spatiotemporal filtering using `mcflirt` (FSL 6.0.5.1:57b01774, Jenkinson et al. 2002). The estimated *fieldmap* was then aligned with rigid-registration to the target EPI (echo-planar imaging) reference run. The field coefficients were mapped on to the reference EPI using the transform. BOLD runs were slice-time corrected to 0.99s (0.5 of slice acquisition range 0s-1.98s) using `3dTshift` from AFNI (Cox and Hyde 1997, RRID:SCR_005927). The BOLD reference was then co-registered to the T1w reference using `mri_coreg` (FreeSurfer) followed by `flirt` (FSL 6.0.5.1:57b01774, Jenkinson and Smith 2001) with the boundary-based registration (Greve and Fischl 2009) cost-function. Co-registration was configured with six degrees of freedom.

Many internal operations of *fMRIPrep* use *Nilearn* 0.8.1 (Abraham et al. 2014, RRID:SCR_001362), mostly within the functional processing workflow. For more details of the pipeline, see [the section corresponding to workflows in *fMRIPrep*'s documentation].

References

Abraham, A., Pedregosa, F., Eickenberg, M., Gervais, P., Mueller, A., Kossaifi, J., Gramfort, A., Thirion, B., & Varoquaux, G. (2014). Machine Learning for Neuroimaging with Scikit-Learn. *Frontiers in Neuroinformatics, 8*(14). https://doi.org/10.3389/fninf.2014.00014

Avants, B. B., Epstein, C. L. , Grossman, M., & Gee, J. C. (2008). Symmetric Diffeomorphic Image Registration with Cross-Correlation: Evaluating Automated Labeling of Elderly and Neurodegenerative Brain. *Medical Image Analysis, 12*(1), 26–41. https://doi.org/10.1016/j.media.2007.06.004

Esteban, O., Blair, R. W., Markiewicz, C. J., Berleant, S. L., Moodie, C., Ma, F., Isik, A. I., et al. (2018). *fMRIPrep* [Software]. https://doi.org/10.5281/zenodo.852659

Esteban, O., Markiewicz, C. J., Blair, R. W., Moodie, C., Isik, A. I., Aliaga, A. E., Kent, J., et al. (2018). fMRIPrep: A Robust Preprocessing Pipeline for Functional MRI. *Nature Methods, 16,* 111-116. https://doi.org/10.1038/s41592-018-0235-4

Gorgolewski, K. J., Burns, C. D., Madison, C., Clark, D., Halchenko, Y. O., Waskom, M. L., & Ghosh, S. (2011). Nipype: A Flexible, Lightweight and Extensible Neuroimaging Data Processing Framework in Python. *Frontiers in Neuroinformatics, 5*(13). https://doi.org/10.3389/fninf.2011.00013

Gorgolewski, K. J., Esteban O., Markiewicz, C. J., Ziegler, E., Ellis, D. G., Notter, M. P., Jarecka, D., et al. (2018). *Nipype* [Software]. https://doi.org/10.5281/zenodo.596855

Jenkinson, M., Bannister, P., Brady, M., & Smith, S. (2002). Improved Optimization for the Robust and Accurate Linear Registration and Motion Correction of Brain Images. *NeuroImage, 17*(2), 825–841. https://doi.org/10.1006/nimg.2002.1132

Jenkinson, M., & Smith, S. (2001). A Global Optimisation Method for Robust Affine Registration of Brain Images. *Medical Image Analysis, 5*(2), 143–156. https://doi.org/10.1016/S1361-8415(01)00036-6

Tustison, N. J., Avants, B. B., Cook, P. A., Zheng, Y., Egan, A., Yushkevich, P. A., & Gee, J. C.. (2010). N4itk: Improved N3 Bias Correction. *IEEE Transactions on Medical Imaging, 29*(6), 1310–1320. https://doi.org/10.1109/TMI.2010.2046908

Section B: EPI distortion correction

Visual demonstration of the EPI susceptibility distortion correction. The images show sagittal sections with the brainstem centered, before and after the correction. The sections are taken approximately at the midline, in close lateral proximity to the left and right LC. The red outline indicates the corrected brain boundary.

**Distorted**	**Corrected**
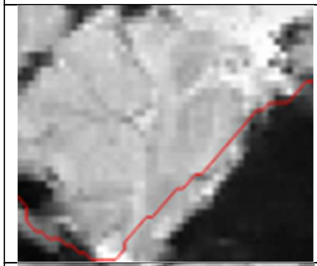	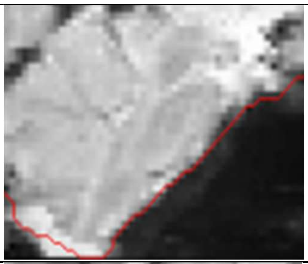
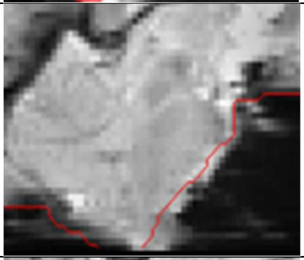	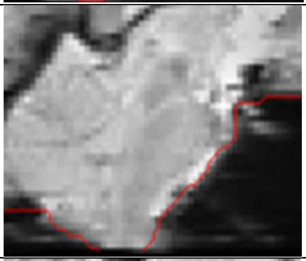
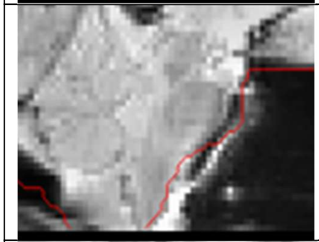	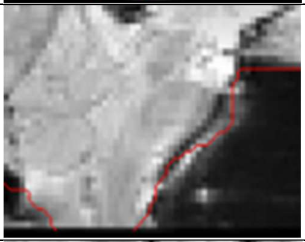
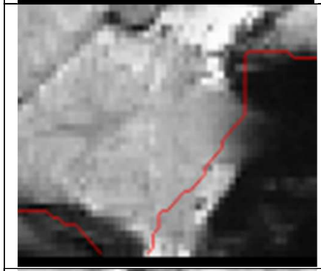	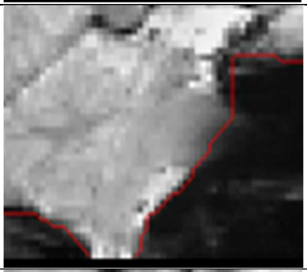
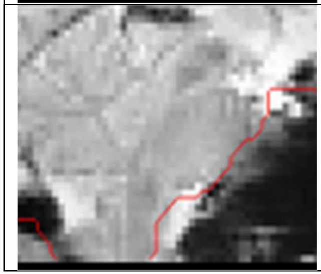	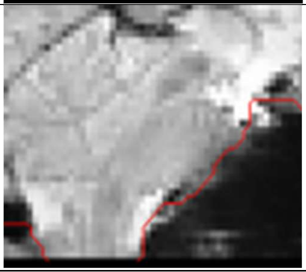

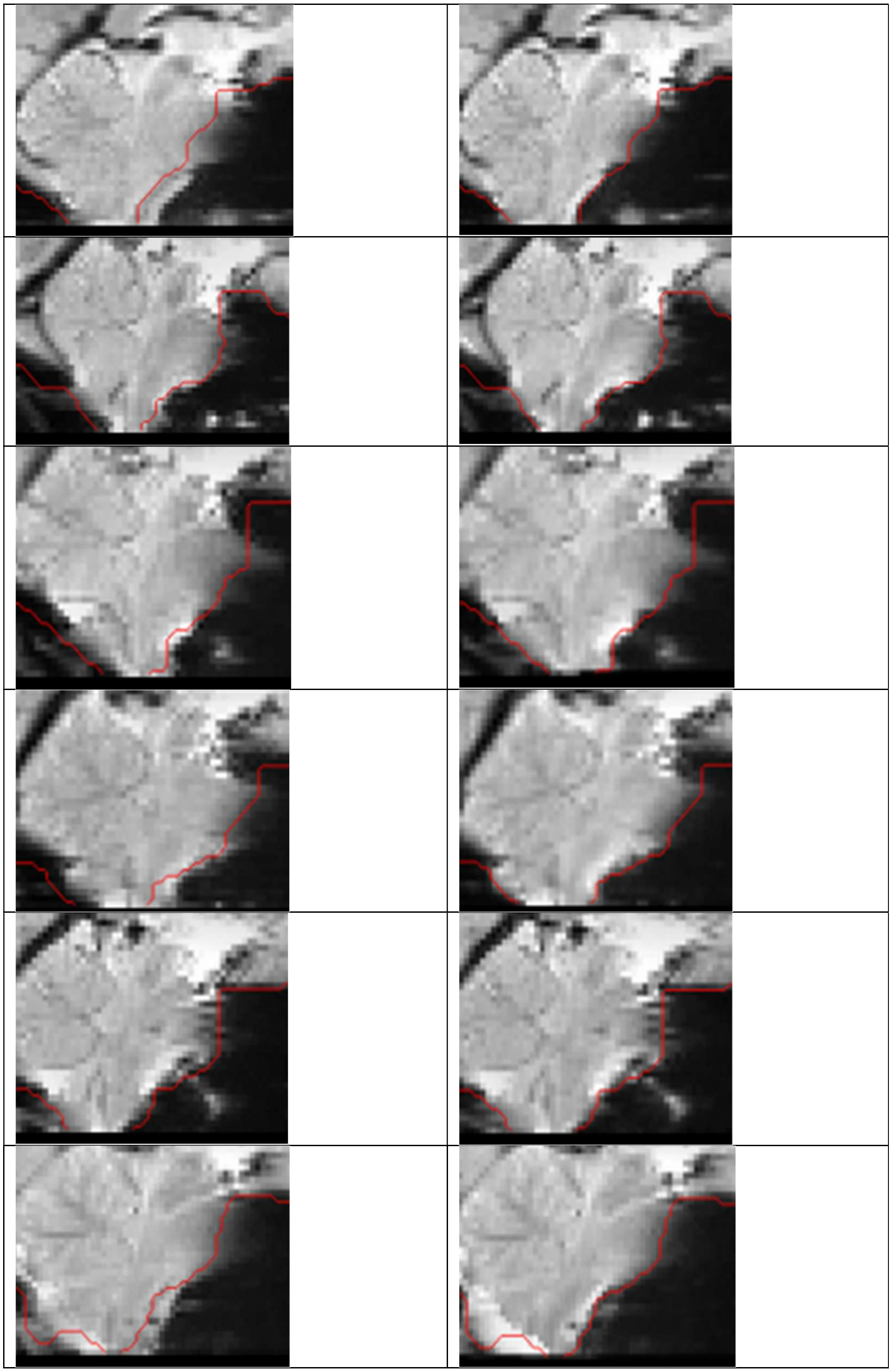

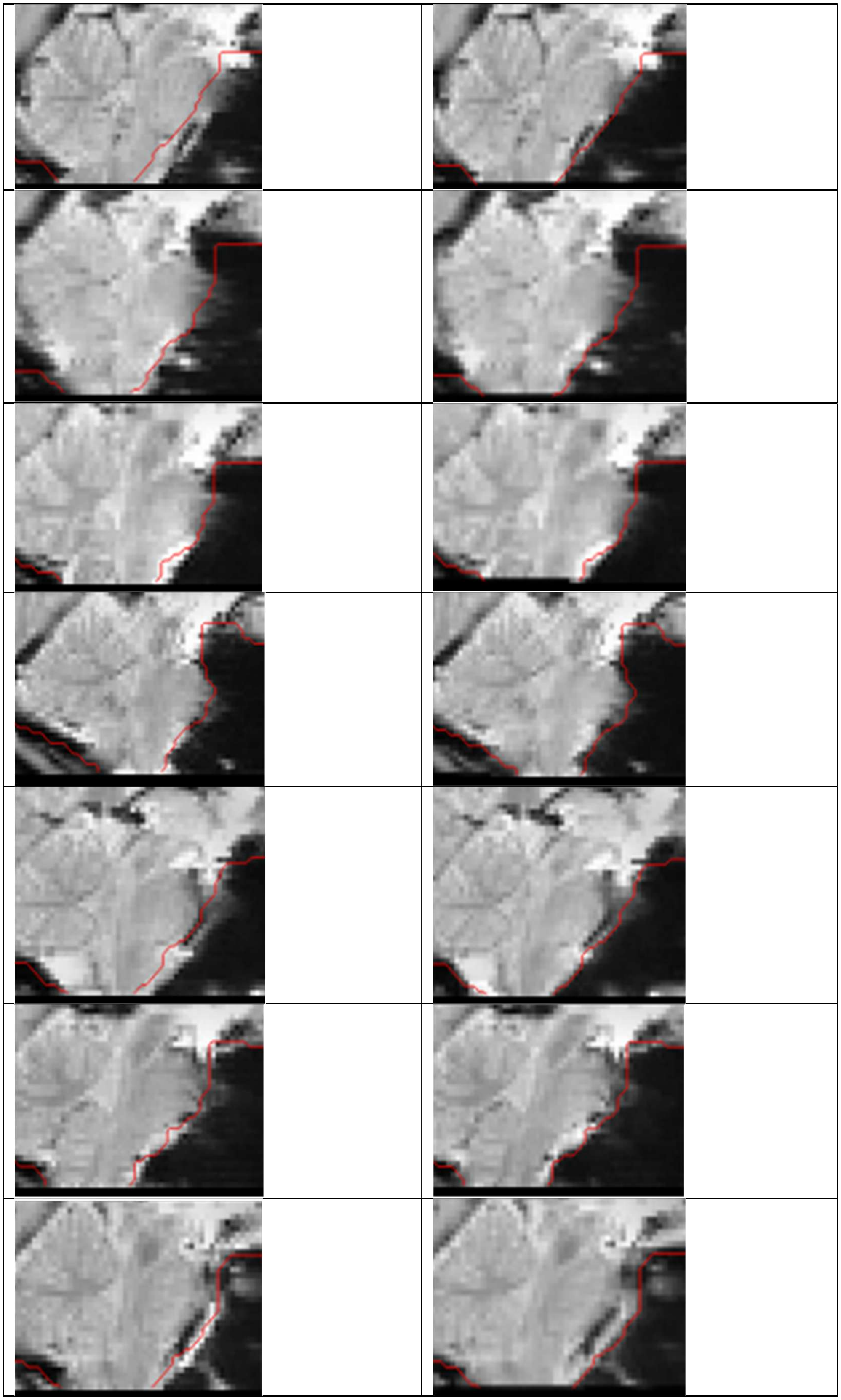

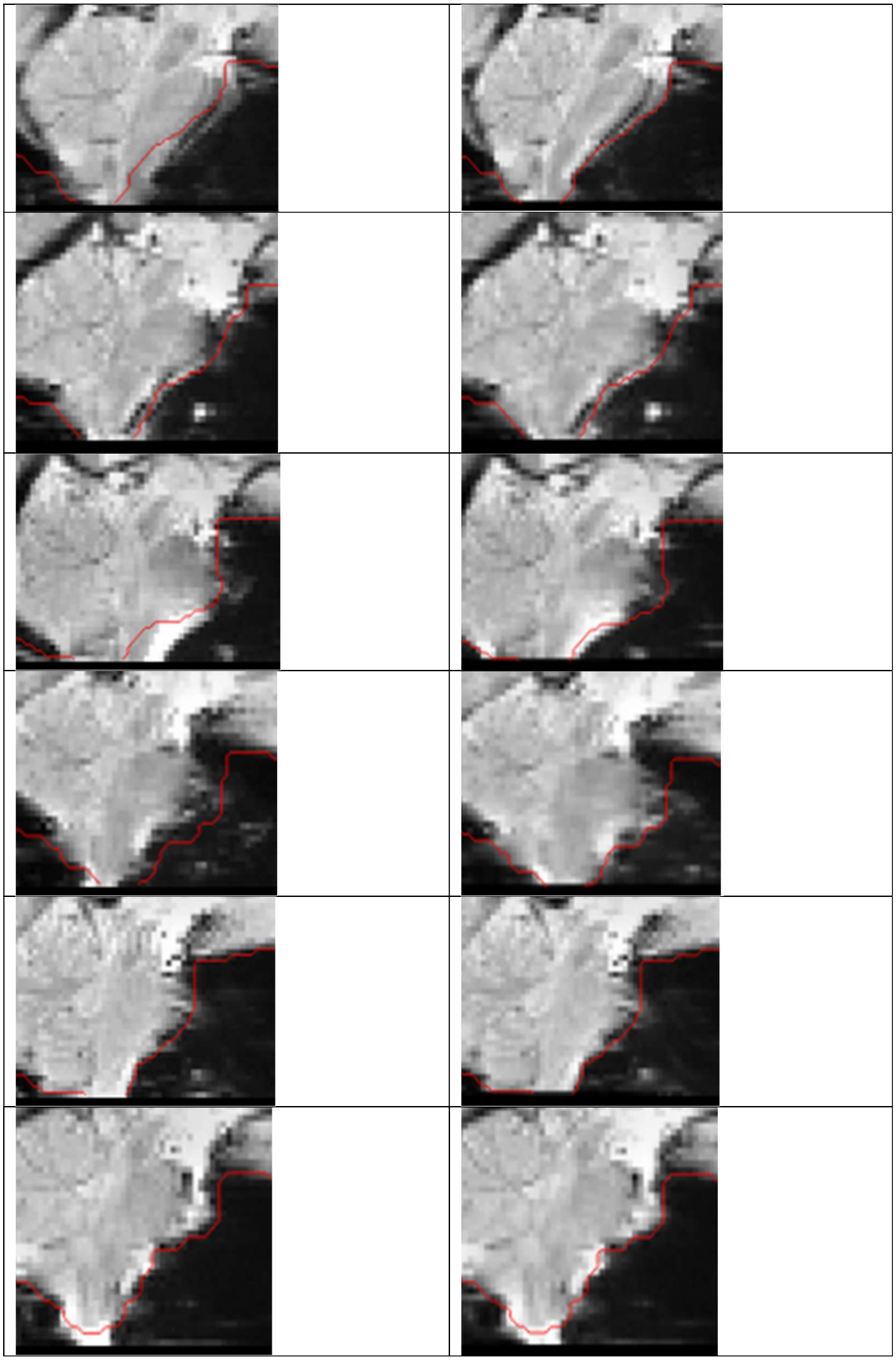

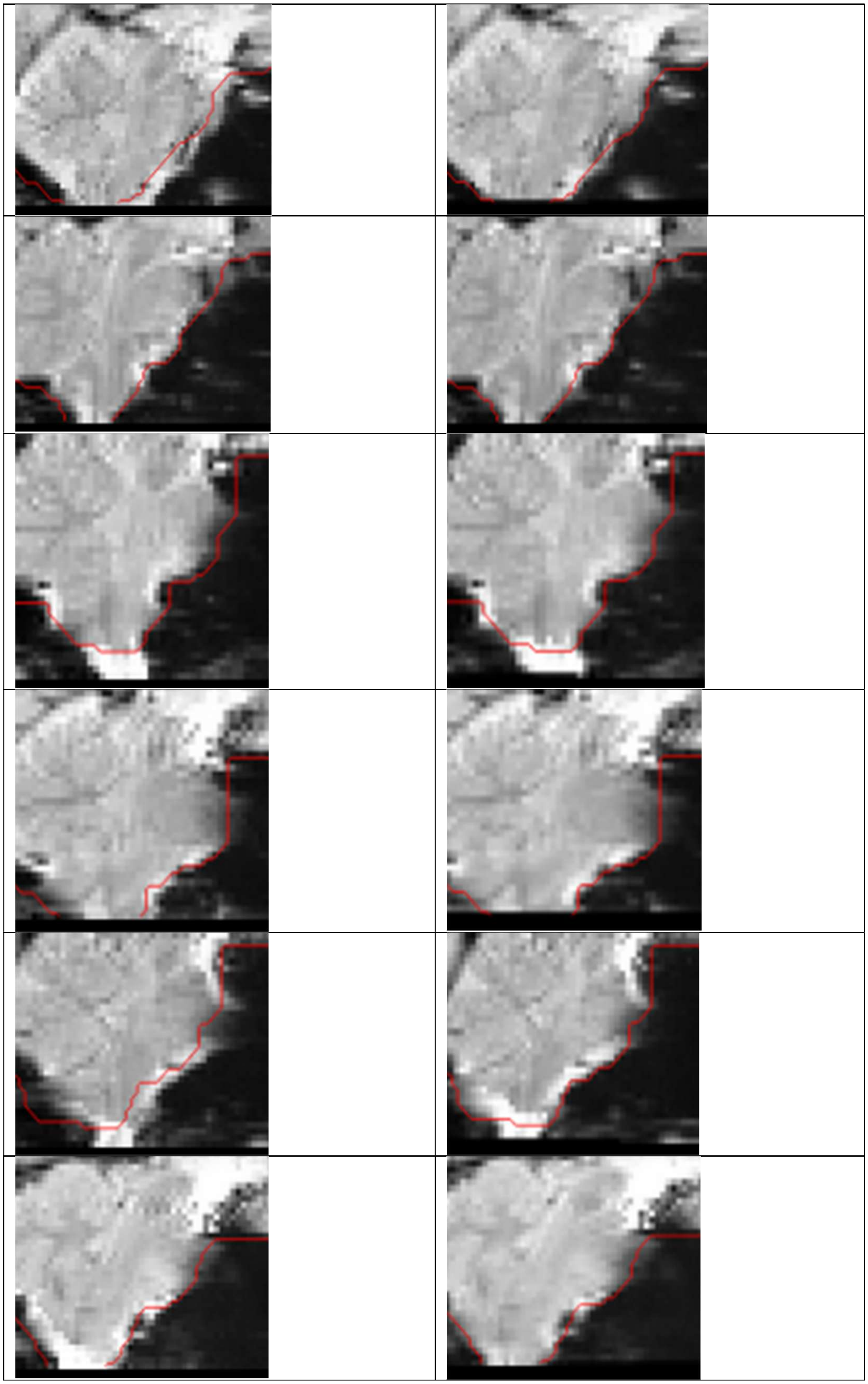

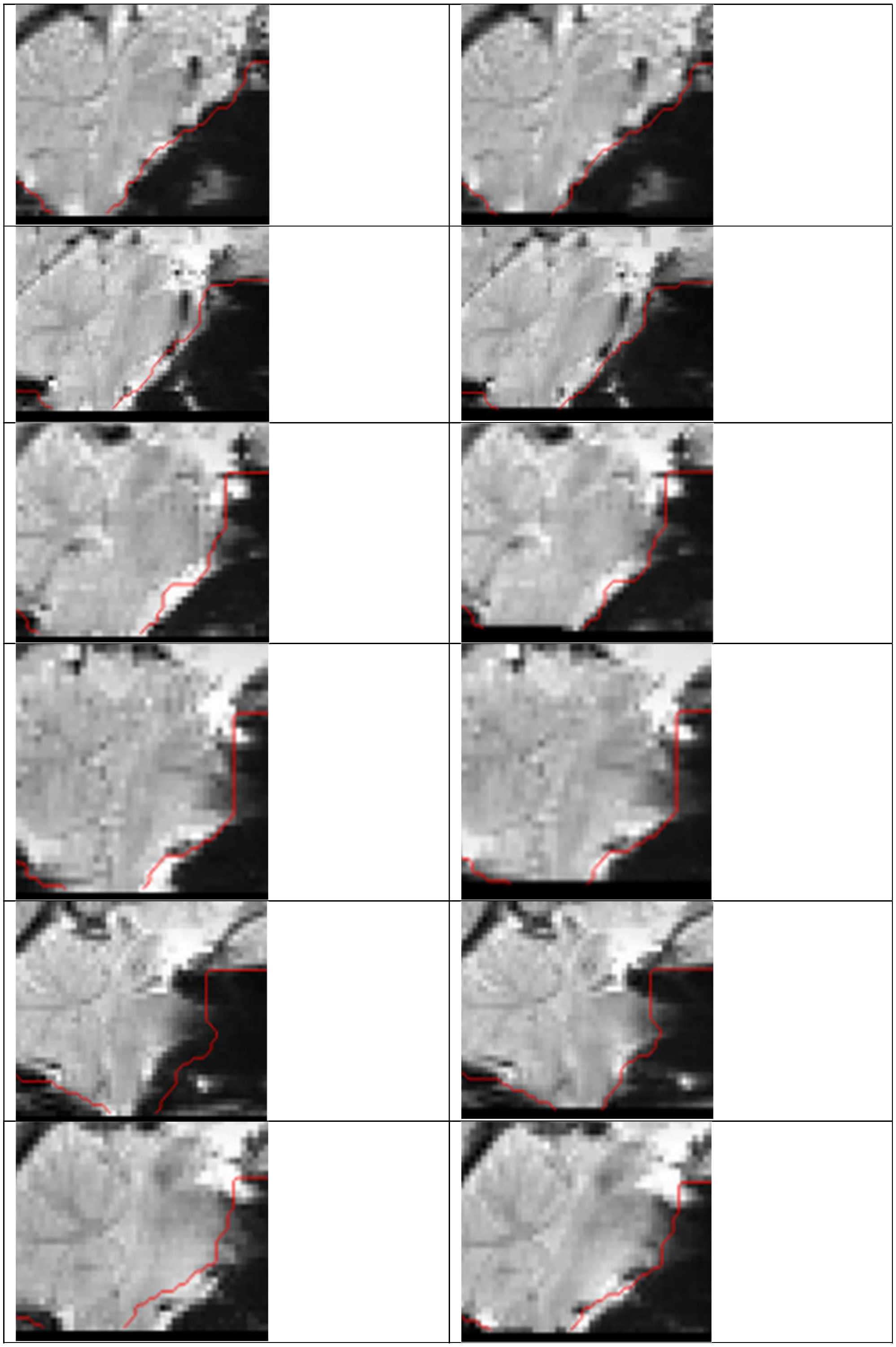

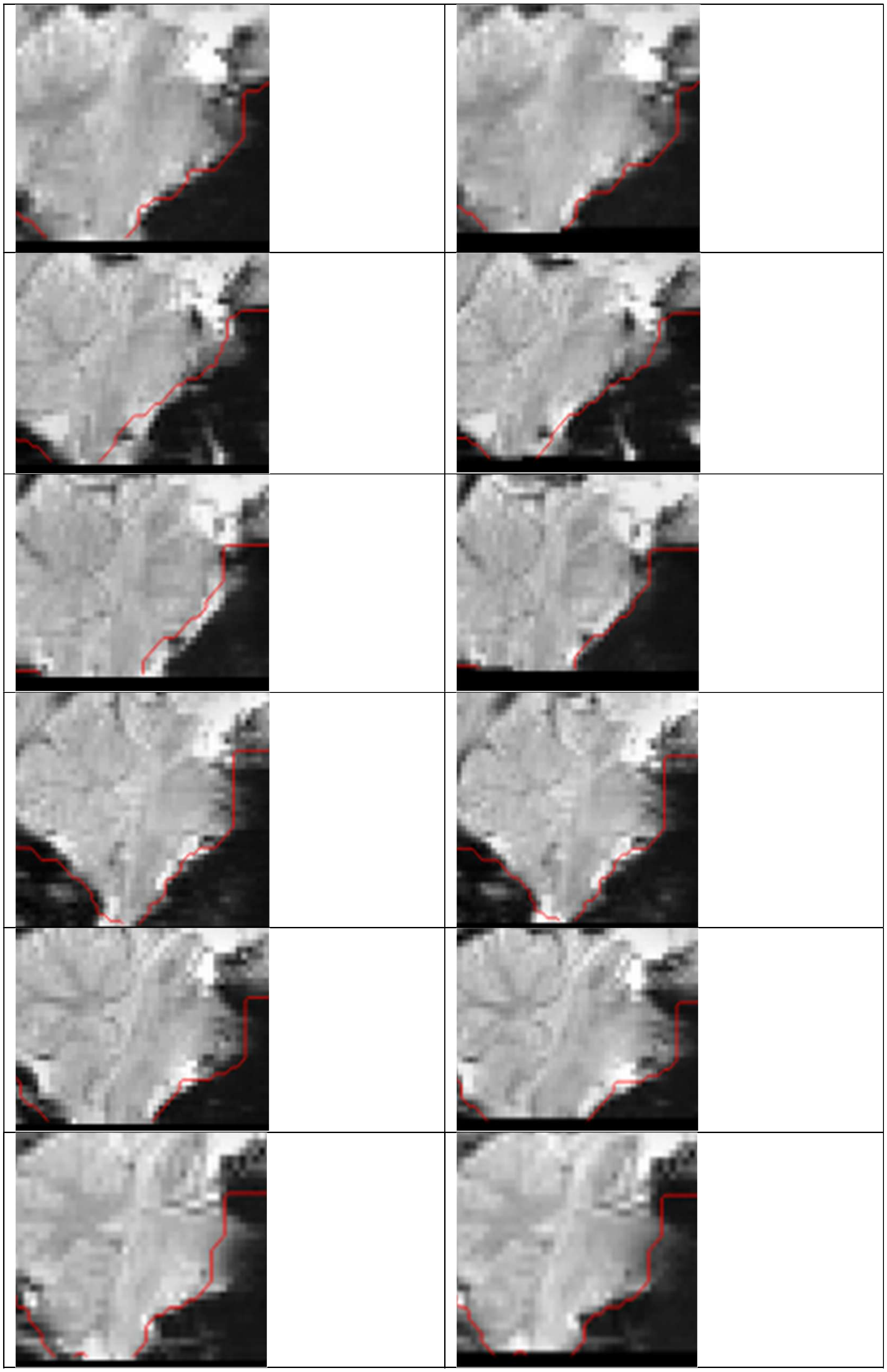

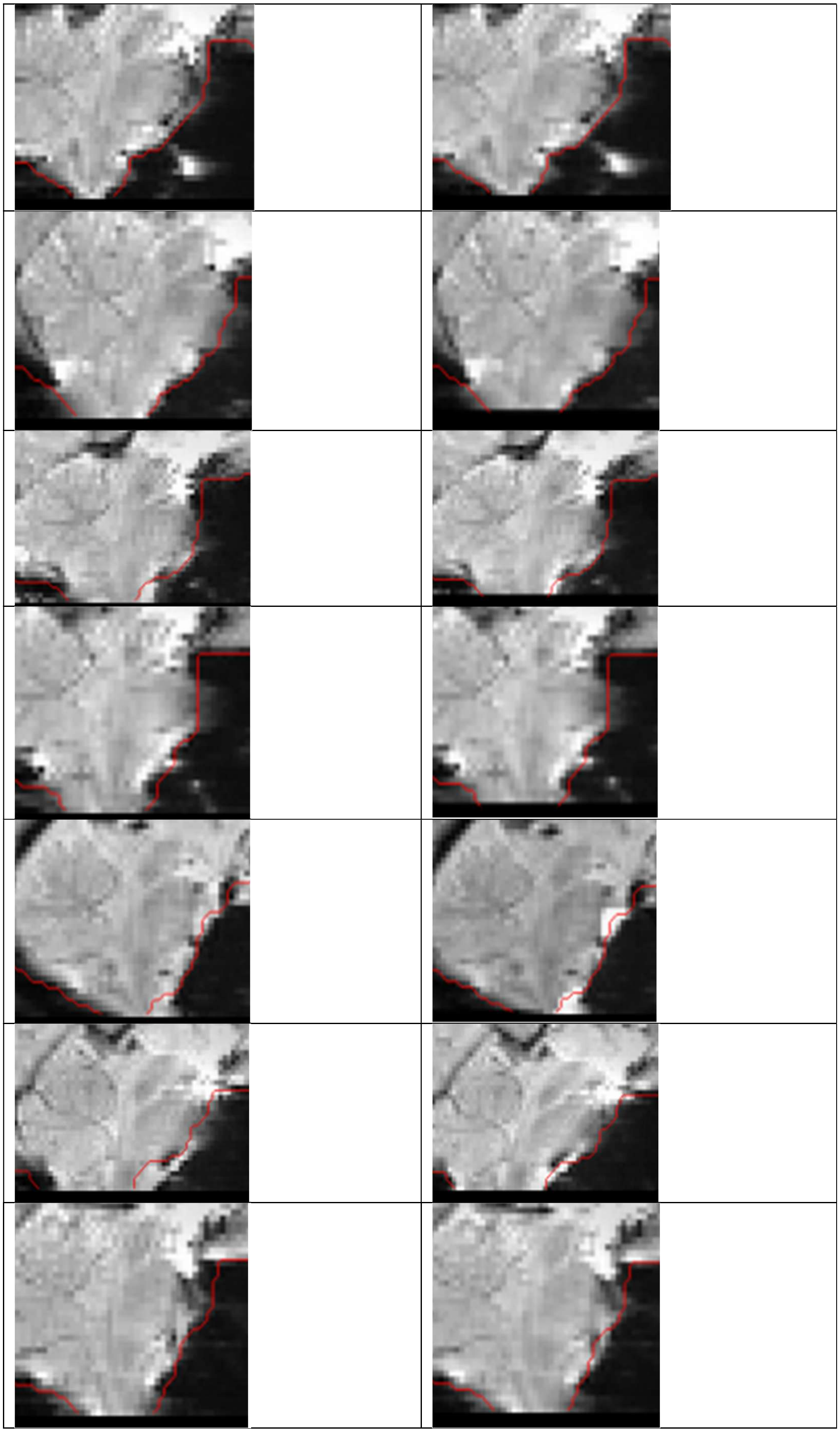

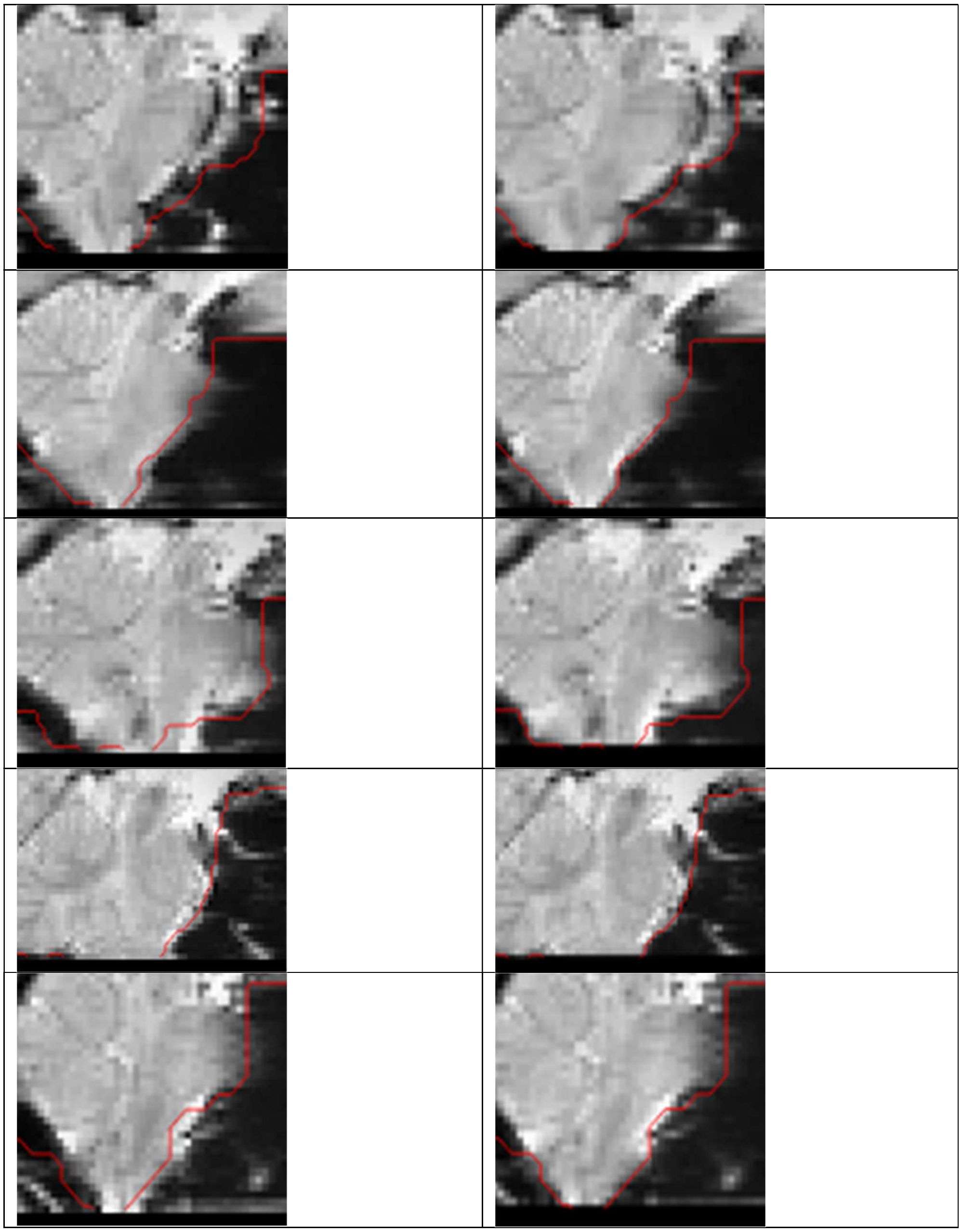

Section C: Individual LC ROI masks

The images below are taken from the horizontal plane of the co-registered TSE scan in native space. Small sections around the 4th ventricle are shown. Note that the LC masks were drawn taking into account the contrast at the typical anatomical location on all three planes.

	**TSE scan (single subject)**	**Same scan with ROI**
**smoothed**	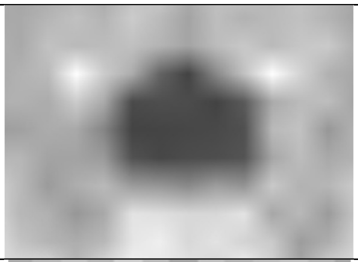	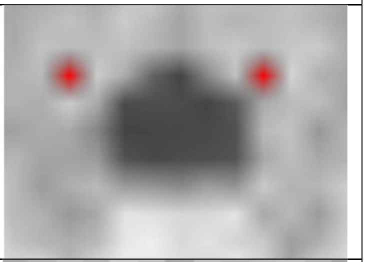
**unsmoothed**	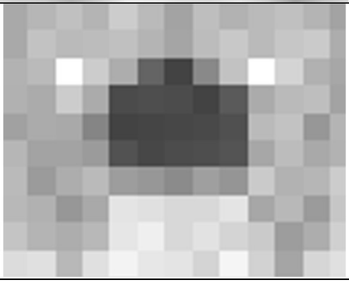	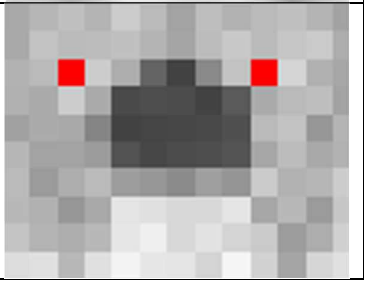

All individual TSE scans with ROI mask

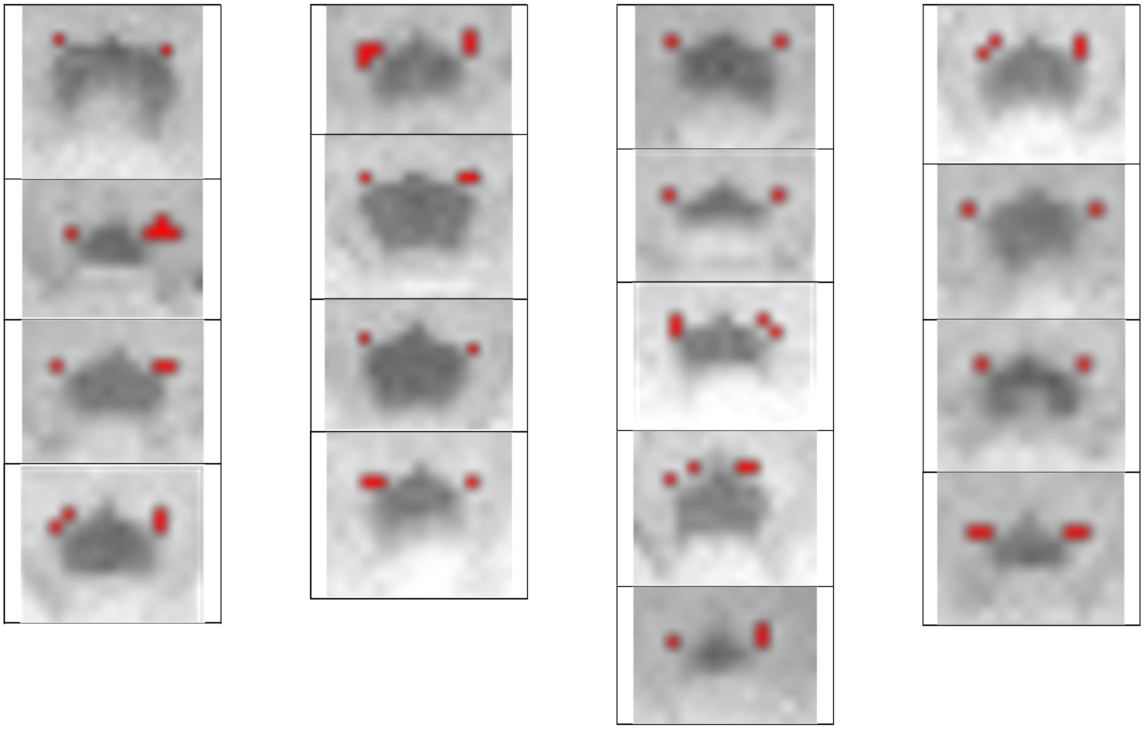

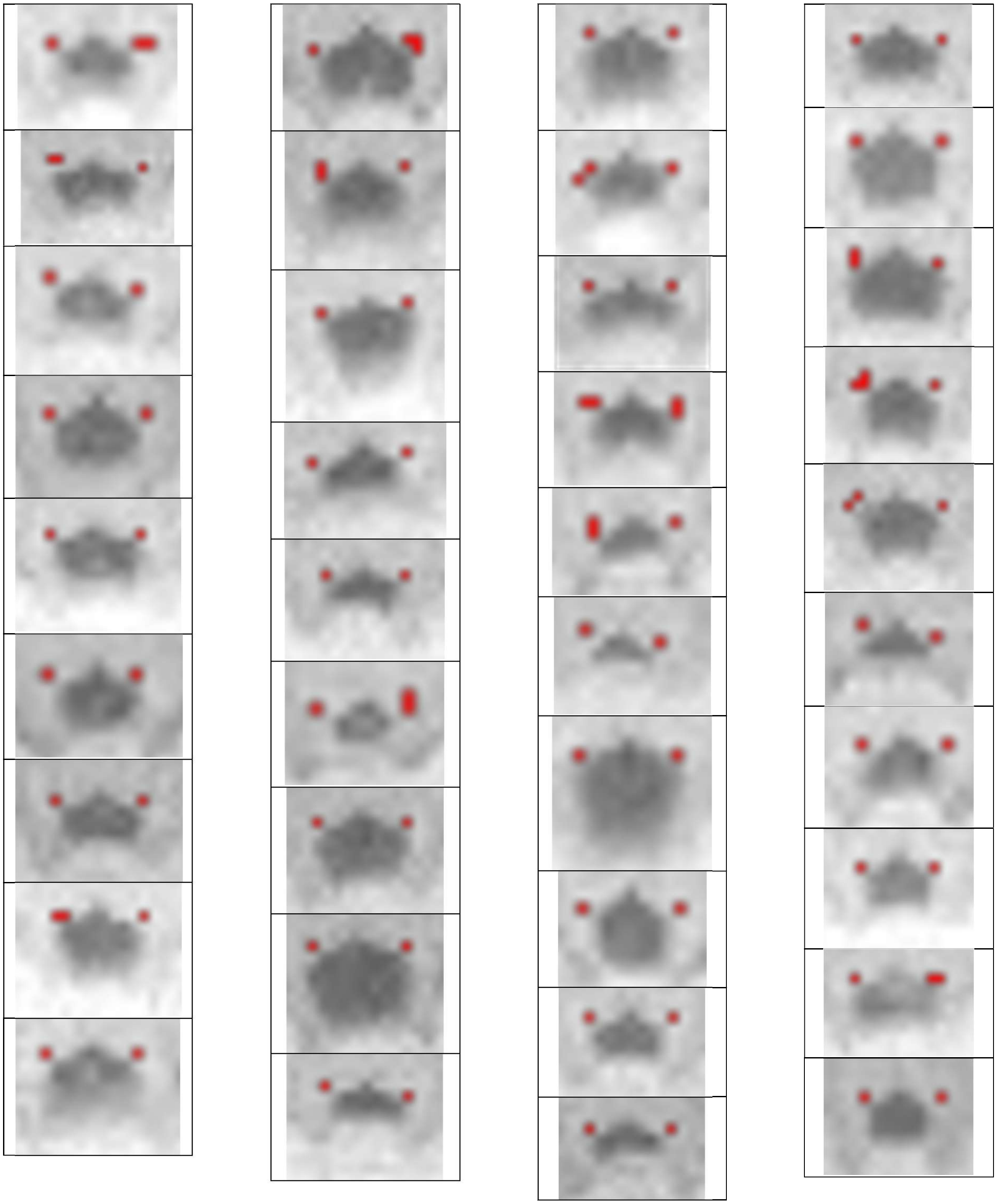

Section D: Post-hoc voxel-wise contrast analysis

As a post-hoc validation that the trialtype effect reflects functional activity at the LC location, and as an additional demonstration of data quality, we conducted a whole-brain voxel-wise contrast analysis comparing target vs. standard trials (target = +1, standard = –1; across event rates). The resulting second-level model (one-sample *t*-test across all participants) indeed revealed activation at the location of the LC when applying a lenient threshold ($p < .001$, uncorrected), with some additional activations which likely reflect noise-related fluctuations rather than systematic effects at this threshold. The application of a strict FWE threshold ($p < 0.05$) resulted in no significant clusters.

The image below shows the relevant section around the LC, with the ROI heatmap across participants shown in blue (identical to Figure 1 in the manuscript), and the statistical map of the contrast with threshold $p < .001$ shown in red.

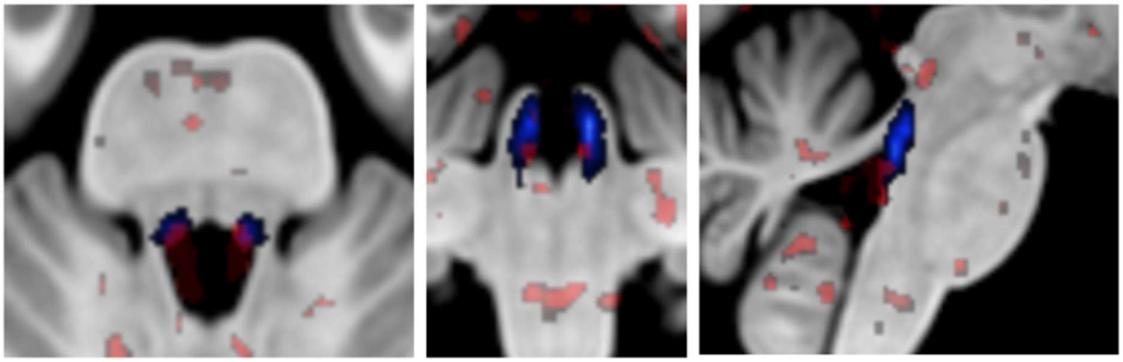

Supplement: Supplementary Material [file IMAG.a.1200_supp.pdf]
